# Excitability-margin narrowing as a candidate gating mechanism for maladaptive circuit reactivation: a ventral CA1-centered model

**DOI:** 10.3389/fnbeh.2026.1839983

**Published:** 2026-04-30

**Authors:** Patryk Rosa

**Affiliations:** Independent Researcher, Warsaw, Poland

**Keywords:** chronic stress, circuit reactivation, excitability margin, hippocampal hyperactivity, major depressive disorder, neuroinflammation, post-traumatic stress disorder, schizophrenia

## Abstract

We propose that the excitability margin (Δ*V*_*margin*_), defined as the difference between spike threshold and resting membrane potential, may function as a quantitative gating variable linking chronic stress, inflammatory load, and transient increases in excitability associated with reactivation to emotionally polarized replay or other maladaptive forms of circuit reactivation. Based on a conceptually guided integration of published electrophysiological data, we modeled how chronic restraint stress, a conservatively parameterized stress-associated inflammatory component, and a transient state of increased engram reactivity may jointly reduce the excitability reserve of ventral CA1 (vCA1) pyramidal neurons. In the main scenario, the model-derived effective margin decreased from 18.4 mV to approximately 6.0 mV, corresponding to a 67.5% reduction. Additional illustrative pro-excitatory scenarios further reduced the margin to approximately 4.3 mV, 3.7 mV, or 1.7 mV. These values fall within the range of physiologically reported amplitudes of transient depolarizing events, including local NMDA spikes, sharp wave–associated depolarizations, intracellular ripples, and larger subthreshold burst-related events. This suggests that in circuits where analogous transient depolarizing events occur, narrowing of Δ*V*_*margin*_ may increase the likelihood that otherwise subthreshold network activity contributes to threshold crossing and maladaptive reactivation. We therefore hypothesize that progressive narrowing of Δ*V*_*margin*_ may act as a gating mechanism for preferential reactivation of vulnerable neuronal ensembles, increasing the probability of repetitive, emotionally polarized replay or other maladaptive forms of circuit reactivation and secondary circuit destabilization. This framework may provide a unifying excitability-based mechanistic hypothesis and a hypothesis-generating transdiagnostic framework relevant to schizophrenia-, depression-, and trauma-related phenotypes, while remaining potentially informative for other conditions characterized by excitability instability. The model generates clear, falsifiable predictions: interventions that widen Δ*V*_*margin*_ or reduce trigger efficacy should attenuate hyperreactivity in the vCA1/vHipp system analyzed here and limit secondary markers of network dysregulation, while also providing a transferable framework for testing analogous low-margin dynamics in other phenotype-relevant circuit nodes.

## Introduction

Schizophrenia, major depressive disorder (MDD), and post-traumatic stress disorder (PTSD) are classified as distinct diagnostic entities; however, they exhibit partial overlap in risk factors and share abnormalities within limbic circuitry. Existing pathophysiological frameworks explain important aspects of these disorders, including aberrant dopaminergic signaling, excitation–inhibition imbalance, hippocampal hyperactivity, stress-axis dysregulation, and neuroinflammatory processes. Yet these accounts are often formulated at different mechanistic levels and do not identify a single quantitative variable linking membrane excitability to the probability of pathological reactivation.

**FIGURE 1:**
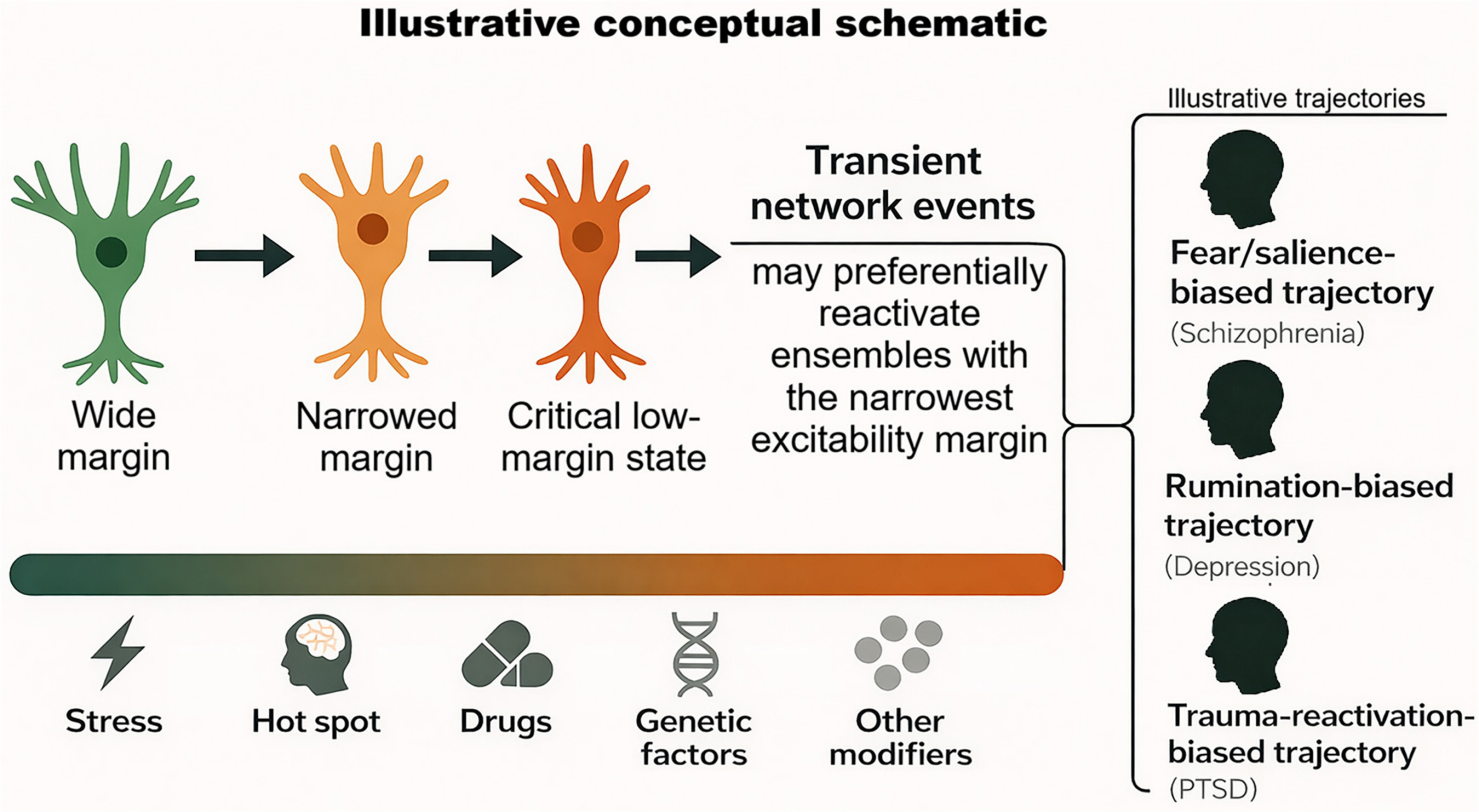
Schematic conceptual overview of the proposed framework. Progressive narrowing of the ventral hippocampal CA1 excitability margin (Δ*V*_margin_) is proposed to increase susceptibility to preferential neuronal ensemble reactivation. Under reduced-margin conditions, transient physiological network events may become more likely to contribute to threshold crossing and reactivation, potentially biasing activity toward fear/ salience-, rumination-, or trauma-reactivation–related trajectories. The figure is intended as an illustrative conceptual schematic and does not represent a linear biophysical simulation of excitability dynamics.

The present framework proposes that narrowing of the excitability margin (Δ*V*_*margin*_) may serve such an integrative role. Within this view, stress- and inflammation-related factors act as upstream drivers of margin reduction, whereas hippocampal hyperactivity, excessive replay, neuromodulatory dysregulation, and longer-term circuit remodeling can be understood as downstream consequences or amplifying processes. The aim is therefore not to replace existing accounts, but to place them within a shared excitability-based gating framework centered on a simple membrane-level quantity–the distance to spike threshold. Unlike broader frameworks centered on hippocampal hyperactivity as a systems-level state, the present model focuses on a membrane-proximal, quantitatively estimable gating variable linking upstream stress- and inflammation-related influences to threshold-near reactivation susceptibility. A graphical overview of the proposed framework is shown in Figure 1.

In the present work, we propose that this variable may be the excitability margin (Δ*V*_margin_), defined as the difference between spike threshold and resting membrane potential:


$$\Delta \,V_{\text{margin}}=V_{\text{thr}}-V_{\text{rest}}$$

This quantity represents the minimal depolarization required to trigger an action potential. In the present manuscript, Δ*V*_margin_ denotes this static distance from rest to spike threshold, whereas Δ*V*_eff_ denotes the functional distance to threshold under active inhibitory and network-state influences. When the margin is wide, physiological oscillations and transient network events remain subthreshold. When it narrows to only a few millivolts, however, physiologically occurring depolarizing events may more readily approach or exceed threshold, thereby increasing the probability of activity initiation. In operational terms, “excitability reserve” refers to the remaining distance to threshold captured by Δ*V*_margin_ and, under inhibitory or network-state conditions, by Δ*V*_eff_.

On this basis, we hypothesize that chronic stress and selected risk factors associated with affective and psychotic disorders may lead to progressive reduction of Δ*V*_margin_ through cumulative effects on resting membrane potential, spike threshold, and overall neuronal excitability. In this framework, margin narrowing is proposed not only as a shared gating variable that increases the probability of threshold crossing during physiological network events, but also as a candidate excitability-based mechanism through which preferential reactivation of previously sensitized neuronal populations may progressively reshape circuit function. If sustained chronically and coupled to replay-dependent plasticity and secondary feedback stabilization, such a state may contribute to phenotype-divergent circuit destabilization from a common excitability-based starting point.

If low-margin states preferentially affect specific cell populations–such as engram cells or elements of frequently recruited affective, mnemonic, or other phenotype-relevant circuits–they may facilitate repeated reactivation of the same network patterns. Over longer time scales, this could promote stabilization of biased activity trajectories which, depending on the dominant circuit content and affective or behavioral salience, may evolve in different phenotype-relevant directions. In this sense, the proposed model does not imply identical downstream disease expression, but rather a shared vulnerability framework in which distinct trajectories diverge from the same excitability-based starting point depending on which circuits become preferentially sensitized and repeatedly reactivated.

As a model node, we focus on pyramidal neurons of the ventral hippocampal CA1 region (vCA1). This choice is motivated by three considerations: first, vCA1 exhibits high sensitivity to chronic stress; second, it maintains strong connectivity with the amygdala and prefrontal cortex–key components of affective circuitry; and third, electrophysiological data are available for this region that allow quantitative estimation of the excitability margin. vCA1 is therefore used here as the principal stress-sensitive and affectively relevant entry point for quantitative analysis, not as an exclusive anatomical claim. More broadly, the framework allows low-margin dynamics to emerge first in different phenotype-relevant circuit nodes, with downstream expression shaped by the location and functional identity of the preferentially reactivated ensembles. In this broader sense, the present vCA1-centered analysis is intended as a region-anchored example of a more general excitability-based vulnerability logic, in which insufficient distance to spike threshold may place selected circuits into a self-reinforcing reactivation-prone state.

The present work develops a phenomenological, semi-quantitative, hypothesis-generating model of Δ*V*_margin_ narrowing in vCA1. To do so, we integrate published electrophysiological data on chronic stress, stress-related inflammatory influences, transient engram reactivation, and selected additional pro-excitatory modifiers, and compare the resulting estimates with amplitudes of physiologically reported transient depolarizing events in order to assess whether normally subthreshold network activity may become more likely to contribute to reactivation.

### Conceptual framework and model assumptions

The present study is based on integration of published electrophysiological data within a phenomenological, semi-quantitative, hypothesis-generating modeling framework. All combined-condition values reported below are model-derived estimates intended to capture order-of-magnitude effects; they were not directly co-measured in the same cells, animals, or experimental preparations and should not be interpreted as precise predictions for a single unified biological system. No new experimental data were generated in this study; all numerical inputs were derived from previously published sources and supplementary model calculations.

#### Definition of the excitability margin

For the purposes of the model, the baseline excitability margin was defined as Δ*V*_margin_ = *V*_thr_ − *V*_rest_, where *V*_thr_ denotes spike threshold and *V*_rest_ denotes resting membrane potential. Under active inhibitory conditions, the effective margin (Δ*V*_eff_) was treated as the functional distance to threshold in the presence of inhibitory conductance influences. For simplicity, changes affecting *V*_rest_, *V*_thr_, or effective inhibition were translated into voltage-equivalent reductions of Δ*V*_margin_.

#### Stress module (CRS)

Stress-induced excitability changes were parameterized using a dimensionless scaling coefficient (γ_rh_) derived from reported reductions in rheobase following chronic restraint stress (MacKenzie and Maguire, 2015): γ_rh_ = I_rh_,_CRS_/I_rh_,_ctrl_. Based on published data, γ_rh_ ≈ 0.56. In the present framework, γ_rh_ was used strictly as a phenomenological compression coefficient for the remaining excitability reserve after stress exposure, rather than as a biophysical conversion from current-domain rheobase values to voltage-domain threshold distance. Its role in the model is therefore operational and integrative: to capture the direction and approximate magnitude of stress-associated margin compression on a shared phenomenological axis.

#### Inflammatory component

A conservative stress-associated inflammatory component was modeled as a depolarizing shift of resting membrane potential: Δ*V*_rest_,_infl_,_stress_ ≈ +2.0 mV. This value corresponds to approximately one-third of the depolarization observed in an acute LPS model (Cheng et al., 2025) and is directionally consistent with evidence supporting a neuroinflammatory component of chronic stress (Calcia et al., 2016; Guo et al., 2014).

#### Engram reactivation “hot spot” module

A transient reduction of the excitability margin during engram reactivation was modeled as: Δ*V*_hot_ = +3.2 mV. This parameter was derived from reported CREB-dependent reductions in spike threshold (Zhou et al., 2009).

#### Cumulative model equation

The final effective excitability margin in the main scenario was computed as: Δ*V*_eff_,_final_ = γ_rh_ (Δ*V*_margin,0_ −Δ*V*_rest_,_infl_,_stress_) −Δ*V*_hot_. Full algebraic derivations, an illustrative local parameter-sensitivity analysis, and alternative parameterizations, including risk-modifier analyses, are provided in Supplementary Methods S1–S6.

### Main modeled scenario

As a reference point, we used a baseline static excitability margin of 18.4 mV for vCA1 pyramidal neurons, defined as the difference between first-spike threshold and resting membrane potential. This value was based on reported electrophysiological properties of ventral CA1 pyramidal cells (Cembrowski et al., 2016). Applying the model parameters specified above (γ_rh_ = 0.56, Δ*V*_rest_,_infl_,_stress_ = +2.0 mV, and Δ*V*_hot_ = 3.2 mV) yielded a model-derived effective margin of approximately 6.0 mV in the main scenario, representing an illustrative reduction from 18.4 mV to ∼6.0 mV (−67.5%). Full assumptions and calculations are provided in Supplementary Methods S1–S4. An illustrative local sensitivity analysis showed that across conservative alternative parameterizations, the qualitative conclusion remained unchanged: Δ*V*_eff_ stayed within a low-millivolt regime rather than returning to a clearly wide-margin baseline state. Depending on the parameter set, the modeled effective margin remained in the ∼5.0–7.0 mV range for one-at-a-time local variation and spanned ∼3.7–8.3 mV across representative combined alternatives (Supplementary Methods S4.7).

### Additional pro-excitatory modifiers

Selected, numerically parameterizable pro-excitatory modifiers were next assessed to determine whether they could further reduce Δ*V*_eff_ relative to the main scenario (∼6.0 mV). Depending on the variant, the resulting effective margin decreased to approximately 4.3 mV (a chronic pro-excitatory component) (Sosulina et al., 2021), 3.7 mV (a stronger inflammatory component) (Cheng et al., 2025), or 1.7 mV (an acute depolarizing effect of caffeine) (Greene et al., 1985). Details are provided in Supplementary Methods S5.

### Comparison to physiologically reported transient depolarizing events

The estimated Δ*V*_eff_ values were then compared with literature-reported amplitudes of transient depolarizing events that could, at least in principle, approach or exceed the remaining margin. The ∼6.0 mV margin is close to the somatic equivalent of local NMDA spikes (5.9 ± 1.5 mV) (Schiller et al., 2000) and lies below part of the reported range of location-dependent NMDA spike/plateau amplitudes (∼3–23 mV) (Major et al., 2008). Sharp wave–associated depolarizations (∼4.1 ± 2.3 mV) (Valero et al., 2015) and intracellular ripple amplitudes (∼1–4 mV) (Ylinen et al., 1995) also fall within the same order of magnitude. Larger subthreshold events have also been reported, including burst-associated depolarizations in the ∼10–25 mV range (Harvey et al., 2009) and place-field subthreshold “hills” of ∼5–20 mV (Epsztein et al., 2011). A consolidated list of values is provided in Supplementary Methods S6.

Overall, these comparisons indicate that as ΔV_eff_ narrows to only a few millivolts, an increasing fraction of physiologically reported transient depolarizing events falls within a voltage range capable of reaching or exceeding the remaining distance to spike threshold. Further narrowing by additional modifiers expands the range of candidate transient events that may cross the effective margin. This comparison is made at the amplitude scale and voltage domain.

## Discussion

The present analysis indicates that the combination of chronic stress, a conservatively modeled inflammatory component, and a transient state of increased engram reactivity may substantially narrow the excitability margin of vCA1 pyramidal neurons. In the main scenario, the modeled margin decreased from 18.4 mV to approximately 6.0 mV, corresponding to a ∼67.5% reduction in baseline excitability reserve. Additional analyses further suggested that selected, numerically parameterizable pro-excitatory modifiers may reduce the margin into the ∼3–4 mV range, and in more extreme variants even to ∼1–2 mV. These values should be interpreted as order-of-magnitude estimates within a phenomenological framework rather than as precise predictions for a single unified biological preparation.

A key implication of this low-margin regime is that physiological transient depolarizing events may become increasingly capable of contributing to threshold crossing and reactivation in susceptible circuits. The model therefore does not invoke a novel form of neural activity; rather, it points to a shift in vulnerability, in which ordinary events that remain subthreshold under a wider excitability reserve may acquire greater pathological relevance when the remaining distance to threshold becomes compressed.

This interpretation should be understood at the level of voltage-domain susceptibility rather than as a full dynamical account of spike initiation or replay generation. In biological circuits, the impact of any given transient event will also depend on temporal structure, dendritic location, conductance state, inhibitory context, and network synchrony.

Although the present discussion focuses on schizophrenia-, depression-, and trauma-related trajectories, the same general logic may be relevant to other disorders characterized by regional hyperexcitability, maladaptive circuit reactivation, and self-reinforcing network destabilization. In this sense, the present work should be understood less as a model of only three clinical phenotypes than as a region-anchored formulation of a broader excitability-based destabilization principle. What may differ across disorders is not necessarily whether low-margin dynamics occur, but where they emerge first, which ensembles become preferentially reactivated, and which downstream molecular, synaptic, and network-level feedback cascades subsequently stabilize the resulting state.

### Population selectivity and preferential reactivation

The consequences of margin narrowing need not be uniformly distributed across the network. If the reduction of Δ*V*_margin_ preferentially affects populations that have been repeatedly recruited in the past–for example, engram cells, ensembles encoding specific emotional, cognitive, or behavioral content, or elements of frequently activated affective, mnemonic, or other phenotype-relevant circuits–the probability of their reactivation may increase more than that of other neurons. This interpretation is consistent with the literature on neuronal allocation, according to which neurons with higher intrinsic excitability are more likely to capture a memory trace, while prior activation and co-allocation can bias subsequent recruitment of related neuronal populations (Josselyn and Frankland, 2018; Pignatelli et al., 2019; Rashid et al., 2016).

In this sense, narrowing of the excitability margin may function not merely as a nonspecific increase in network excitability, but as a mechanism that selectively privileges particular activity traces. Repetitive reactivation would therefore not need to produce global hyperactivity across the entire system; gradual strengthening of specific loops sharing common emotional, cognitive, or behavioral content could be sufficient to progressively bias network dynamics over time.

### From margin narrowing to phenotypic trajectories

If different circuit types become preferentially affected, downstream network consequences may diverge into phenotype-specific directions. In this sense, narrowing of Δ*V*_margin_ is proposed as a shared excitability-based starting point from which distinct circuit architectures, dominant contents, and downstream feedback cascades may give rise to different phenotype-relevant trajectories. The schizophrenia-, depression-, and trauma-related directions outlined below should therefore be understood as illustrative trajectory examples rather than as an exhaustive boundary of the model.

If fear-/salience-related circuits become preferentially engaged, a chronically reduced margin may promote a fear-/salience-biased trajectory resembling psychosis-related components. In the schizophrenia literature, increased amygdala reactivity to neutral stimuli, disruptions of θ–γ coupling, excitation–inhibition imbalance, and vulnerability of PV/PNN systems–particularly under oxidative stress conditions–have been described (Cabungcal et al., 2013; Hall et al., 2008; Koutsoukos et al., 2013; Mauney et al., 2013; Rowland et al., 2013; Uhlhaas and Singer, 2010). Within this context, stabilized preferential reactivation of fear-/salience-related circuits could secondarily bias processing toward limbic dominance at the expense of cortical control.

If, instead, circuits associated with negative valence, self-referential processing, and rumination become preferentially stabilized, a similar mechanism may manifest as a sadness-/rumination-biased trajectory consistent with depressive phenotypes. The MDD literature reports increased coupling of the subgenual anterior cingulate cortex (sgACC) with the default mode network (DMN), relative dominance of the DMN over task-positive networks, alterations in GABA/Glx balance within the ACC, dysregulation of the CRF/HPA axis, and progressive structural and synaptic changes in the prefrontal cortex under chronic stress (Godfrey et al., 2018; Hamilton et al., 2011, 2015; Kang et al., 2012; Kassem et al., 2013; Nemeroff et al., 1984; Ogawa et al., 2018). Within this framework, a persistently reduced excitability margin could increase the likelihood of repeated reactivation of negative-affect loops, thereby promoting stabilization of a ruminative activity pattern.

In trauma-related circuits, the same mechanism may reinforce a trauma-reactivation–biased trajectory consistent with PTSD phenotypes. The literature indicates that trauma recall and trauma-related cue exposure strongly engage limbic and stress systems. One plausible amplification mechanism is the locus coeruleus (LC) → basolateral amygdala (BLA) projection, associated with increased noradrenergic drive, alongside reported increases in CRF signaling and region-specific GABAergic–glutamatergic abnormalities in cortico-limbic nodes (Bremner et al., 1997; Hughes and Shin, 2011; McCall et al., 2017; Rosso et al., 2014, 2017; Shin and Liberzon, 2010). Under such conditions, repetitive preferential reactivation of trauma-associated ensembles could facilitate further consolidation of intrusive activity patterns.

A shared element across the trajectories developed in the present work may be gradual convergence onto local high-excitability “hot spots” characterized by strengthened synaptic connectivity. In particular, the vCA1 → basolateral amygdala pathway, embedded within the broader hippocampal–amygdalar network, represents a plausible convergence node in the phenotype-related directions analyzed here. Aversive learning enhances synaptic plasticity within hippocampus–amygdala connections, and engram studies indicate that neurons with higher intrinsic excitability are preferentially recruited, co-allocated, reactivated, and progressively stabilized through synaptic mechanisms during subsequent related experiences (Clem and Huganir, 2010; Kim and Cho, 2020; Kitamura et al., 2017; Rao-Ruiz et al., 2021; Rumpel et al., 2005; Ryan et al., 2015; Yiu et al., 2014).

Importantly, a chronically reduced excitability margin may influence not only reactivation of existing traces but also–at least in circuits in which engram-allocation mechanisms and partial representational overlap play an important role–the allocation of new experiences. Consistent with the literature showing that more excitable neurons are preferentially recruited into engrams, and that temporally proximal events may be represented by partially overlapping neuronal populations (Cai et al., 2016; Josselyn and Frankland, 2018; Rashid et al., 2016), a persistently low Δ*V*_margin_ could increase the probability that newly encoded experiences become partially incorporated into already privileged ensembles. In the context of the present vCA1-centered model, this would provide a biologically plausible route by which initially selective reactivation trajectories could broaden, stabilize, and acquire increasingly complex or partially mixed symptom content.

Although the present discussion focuses on schizophrenia-, depression-, and trauma-related trajectories anchored in vCA1-centered circuitry, the same general logic may also be testable in other conditions in which low-margin dynamics emerge in different phenotype-relevant circuit nodes. A full elaboration of the phenotype-specific trajectories developed here is provided in Supplementary Discussion SD1–SD4, whereas the broader interpretation of the model in terms of a regional “ignition-site” framework is discussed in SD4.7.

### Potential feedback mechanisms sustaining a low-margin state

One possible consequence of repeated reactivation of preferentially recruited neuronal populations is the emergence of positive feedback loops that not only increase the likelihood of subsequent activations but also contribute to stabilization of the reduced excitability reserve itself. In this framework, narrowing of Δ*V*_margin_ would not represent merely a transient consequence of stress or engram reactivation, but could evolve into a partially self-sustaining network state.

A first candidate mechanism is a stress–glucocorticoid–memory feedback loop. Stress activates the hypothalamic–pituitary–adrenal (HPA) axis and increases glucocorticoid levels, and existing literature indicates that cortisol modulates memory retrieval and stress-related memory bias (Schönfeld et al., 2014; Wolf, 2017). In practical terms, frequent reactivation of emotionally charged circuits may co-occur with a hormonal environment that further modifies reactivation thresholds and selectively privileges the same memory traces, thereby reinforcing their future accessibility.

A second candidate mechanism involves stress–inflammation coupling affecting chloride-dependent inhibition. Experimental data indicate that chronic stress may weaken KCC2 function and shift *E*_GABA_ in a depolarizing direction, thereby increasing CA1 neuronal excitability (MacKenzie and Maguire, 2015). At the same time, both experimental and review literature suggest that pro-inflammatory signaling can modulate the NKCC1/KCC2 balance and disrupt chloride homeostasis; cytokines such as IL-1β and IL-6 signaling have been associated with altered chloride transporter regulation and increased excitability (Kalkman, 2019; Pozzi et al., 2020). In this sense, a chronically reactivated stress–inflammatory state could further stabilize a low excitability margin through secondary weakening of GABAergic inhibition.

A third candidate mechanism concerns redox imbalance, PV interneurons, perineuronal nets (PNNs), and network synchrony. Translational studies indicate that glutathione deficits and oxidative stress particularly burden fast-spiking parvalbumin interneurons, while PNNs serve protective functions for these cells (Cabungcal et al., 2013; Steullet et al., 2017). If repeated reactivation and associated metabolic or redox stress promote further dysfunction of PV interneurons and degradation of PNNs, inhibitory control and network synchronization may deteriorate secondarily. This interpretation is directionally consistent with literature linking PV interneuron dysfunction to beta/gamma oscillatory disturbances and reduced synchrony in schizophrenia (Uhlhaas and Singer, 2010).

Additional interacting processes–including dopamine–salience feedback, maladaptive homeostatic plasticity, astrocyte-mediated glutamate dysregulation, and metabolic or mitochondrial stress–may also contribute to stabilization of the low-margin state, insofar as they may further increase membrane excitability, weaken inhibitory control, disrupt ionic homeostasis, or reinforce network susceptibility to reactivation; these processes therefore warrant further investigation (Chen et al., 2022; Howes and Nour, 2016; Kätzel et al., 2020; Kruyer et al., 2023; Lodge and Grace, 2011; Norat et al., 2020; Perez and Lodge, 2013; Todd and Hardingham, 2020).

Taken together, these considerations support a two-layer model. The first layer consists of the quantitatively defined narrowing of Δ*V*_margin_ demonstrated in the present work. The second layer comprises qualitatively described feedback mechanisms related to stress, inflammation, redox imbalance, and other secondary processes that may sustain and amplify this state over time. By weakening chloride-dependent inhibition, impairing PV interneuron–dependent timing control, and increasing metabolic and oxidative burden, these second-layer processes may further reduce effective inhibitory restraint, destabilize threshold control, and thereby promote consolidation of the low-margin state. Such a framework preserves the simplicity of the quantitative core while at the same time providing biologically plausible pathways through which repeated reactivation may evolve into an increasingly stable, self-reinforcing state of heightened reactivation susceptibility.

If sustained chronically, such low-margin dynamics would be expected to influence not only reactivation susceptibility and network synchrony, but also broader functional domains including cognitive bias, emotional regulation, replay-dependent synaptic plasticity, and–in selected contexts–neurodegenerative vulnerability. In this sense, the proposed framework may also help explain how repeated threshold-near reactivation could propagate beyond local excitability changes and lead to more persistent impairments of network control and adaptive behavioral regulation.

### Predicted classes of interventions and combination strategies

Within the proposed framework, it is useful to distinguish between interventions acting at different mechanistic levels of the low-margin state. The most direct class includes interventions that widen Δ*V*_margin_ itself, whereas additional classes may reduce trigger efficacy, restore inhibitory timing and synchrony, strengthen top-down control, weaken replay-dependent reinforcement, or interrupt feedback processes that help stabilize a chronically reduced excitability reserve.

The first class includes manipulations that increase the distance between spike threshold and resting membrane potential–for example, restoration of a more hyperpolarized resting potential, a less depolarizing *E*_GABA_, increased rheobase, or improved chloride homeostasis through enhanced KCC2 function and reduced NKCC1 predominance. Such interventions would be expected to directly decrease circuit susceptibility to reactivation, consistent with literature identifying KCC2 as a promising therapeutic target in states of neuronal hyperexcitability and excitation–inhibition imbalance (Duy et al., 2020; Kadam and Hegarty, 2024).

The second class comprises interventions that reduce the effective amplitude or convergence of triggering events without necessarily widening Δ*V*_margin_ itself. One candidate mechanism is improved phase control mediated by parvalbumin (PV) interneurons and more physiologically coordinated gamma oscillations. In this view, enhanced inhibitory synchronization would reduce the temporal overlap of excitatory inputs onto pyramidal cells and thereby decrease the number of convergent events capable of exceeding the remaining margin (Hijazi et al., 2023; Sohal et al., 2009; Wirk et al., 2025).

A third class includes interventions that attenuate NMDA- and Ca^2+^-dependent amplification processes contributing both to strong depolarizing events and to plastic strengthening of reactivated traces. Such manipulations would be predicted to weaken secondary engram strengthening, reduce reconsolidation, and limit the transition from single reactivation events to persistent positive feedback loops (Bauer et al., 2002; Crestani et al., 2015; Da Silva et al., 2013).

A fourth class includes interventions that strengthen top-down cortical regulation of limbic replay-prone circuits, thereby reducing repeated amplification of vulnerable hippocampal–amygdalar activity patterns and limiting the behavioral dominance of emotionally polarized reactivation trajectories (Delgado et al., 2008; Lei et al., 2024; Likhtik and Paz, 2015; Plas et al., 2024).

A fifth class includes interventions that weaken replay-dependent reconsolidation and preferential reallocation of activity into already sensitized ensembles. Because repeated reactivation may strengthen and broaden privileged activity traces over time, reducing reconsolidation efficacy or limiting incorporation of newly encoded material into the same ensembles would be predicted to constrain progressive stabilization of maladaptive loops (Borgomaneri et al., 2020; Cai et al., 2016; Haubrich and Nader, 2016; Josselyn and Frankland, 2018; Sierra et al., 2023).

A sixth class includes interventions that interrupt stress–inflammatory–redox–autonomic feedback processes that may help sustain the low-margin state over time, including inflammatory modulation of chloride homeostasis, stress-linked hippocampal inflammation, and redox-related impairment of PV interneuron function (Calcia et al., 2016; Liu et al., 2024; Namgung et al., 2022; Pozzi et al., 2020; Steullet et al., 2017).

In practical terms, the model predicts that these intervention classes should converge on a shared functional outcome: reduced susceptibility to maladaptive reactivation, attenuated hyperreactivity in the vCA1/vHipp system analyzed here, and weakening of the secondary processes that sustain pathological network states. More broadly, the framework predicts that, in other phenotype-relevant circuit nodes, analogous interventions should reduce local hyperreactivity, limit secondary network destabilization, and weaken the feedback processes that stabilize the low-margin state. The model further predicts that combination strategies acting at more than one mechanistic level may produce stronger or more durable effects than single-node interventions, because pathological threshold crossing depends jointly on excitability reserve, transient input structure, and feedback stabilization of repeated reactivation.

### Expected direction of network- and neuromodulation-level changes

The model further implies that an effective intervention–whether it acts by widening the excitability margin or by reducing trigger efficacy–should produce a recognizable directional shift in secondary network-level markers. The primary expected effect would be reduced circuit hyperreactivity, including less frequent engram reactivation, decreased vCA1 hyperactivity, weaker spike coupling to fast network events, and more physiologically coordinated limbic–cortical synchrony. Secondarily, such changes would be expected to attenuate stress-, inflammatory-, and redox-related feedback processes that help sustain the low-margin state, thereby favoring normalization of excitation–inhibition balance, HPA-axis burden, and PV/PNN vulnerability.

In psychosis-like phenotypes, this should not be interpreted as a simplistic linear effect such as “increasing the margin always lowers dopamine.” Rather, the more precise prediction is that reducing hippocampal hyperreactivity should attenuate upstream drive onto the dopaminergic system and thereby favor normalization of dopamine neuron activity and aberrant salience attribution, consistent with previous psychosis models (Kätzel et al., 2020; Lodge and Grace, 2011; Perez and Lodge, 2013).

Analogously, where low-margin states are sustained by stress–inflammatory coupling, disrupted chloride homeostasis, or other phenotype-relevant secondary mechanisms, successful intervention would be expected to shift the system toward lower stress-axis load, a weaker pro-inflammatory milieu, improved GABAergic control, or a more stable network state. These changes need not be identical across phenotypes, but they should be directionally consistent with reduced susceptibility to self-sustaining reactivation.

### Model limitations

The present model is integrative and phenomenological in nature. Its primary strength lies in reducing heterogeneous electrophysiological findings to a shared quantitative axis defined by Δ*V*_margin_; however, this same feature also constitutes its principal limitation. The parameters incorporated into the model derive from different preparations, species, experimental paradigms, and biological contexts, and therefore do not represent a single directly measured parameter set obtained from one brain, one neuronal population, and one unified physiological state. In addition, not all input parameters were obtained directly from vCA1; some were extrapolated from general CA1 or from other pyramidal neuron populations. Accordingly, the model should be interpreted as a quantitatively anchored hypothesis regarding the order of magnitude of the effect, rather than as a precise numerical description specific to human vCA1.

A second limitation is that the model does not constitute a full biophysical description of the neuron, but rather a simplified functional approximation. In particular, the coefficient γ_rh_ was treated as an operational index of excitability reserve compression rather than as a direct surrogate for a specific ion-channel parameter or a single membrane mechanism. Similarly, several additional modifiers were translated into voltage-equivalent terms based on experimentally observed effects. Although this allows comparison along a shared quantitative axis, it does not capture the full nonlinearities of real synaptic integration and membrane dynamics. Relatedly, the trigger analysis is based on amplitude-scale comparisons rather than on full dynamical network simulations. The framework therefore identifies conditions under which threshold crossing becomes more plausible in voltage terms, but does not in itself simulate temporal structure, dendritic location, conductance state, inhibitory context, synchronization, local connectivity architecture, or state-dependent network variability, and thus does not by itself demonstrate spontaneous replay, prove functional equivalence between transient amplitude and spike-triggering efficacy, or define a single universal “pathology threshold.”

An additional limitation is the absence of direct experimental validation of Δ*V*_margin_ as a predictive biomarker of pathological reactivation. Establishing whether this quantity prospectively predicts reactivation susceptibility remains a central empirical task for future work.

Finally, the proposed phenotype-specific trajectories (fear-/salience-biased, sadness-/rumination-biased, trauma-reactivation–biased) should be understood as directionally literature-consistent mechanistic sketches rather than as complete experimentally established causal chains. Their purpose is to illustrate how a shared gating variable–narrowed excitability margin–could be embedded within distinct circuit and emotional contexts.

### Future research directions

Future studies should directly test whether engram and non-engram cells differ in Δ*V*_margin_, resting membrane potential, first-spike threshold, and rheobase under stress conditions and following interventions aimed at restoring excitability reserve. A first stringent test would be a multi-axis *ex vivo* or *in vitro* paradigm designed to model cumulative low-margin states and their rescue. For example, vCA1 pyramidal preparations could be assigned to control, single-hit, combined-hit, single-axis rescue, and combined-rescue conditions in order to determine whether convergent stress-, chloride-, and reactivation-related manipulations narrow Δ*V*_margin_ more strongly than single manipulations, and whether multi-axis rescue widens the margin more effectively than single-axis interventions. Readouts could include *V*_rest_, *V*_thr_, rheobase, Δ*V*_margin_, chloride-related inhibitory shifts, and Ca^2+^-linked trigger amplification.

A second critical test would be an activity-tagged *in vivo* mouse paradigm combining stress exposure, identified engram-cell populations, and intervention-based rescue. In such a design, stress-related manipulations would be predicted to reduce Δ*V*_margin_ most clearly in reactivation-prone or tagged neuronal ensembles, increase vCA1/vHipp hyperactivity, and enhance coupling to transient depolarizing events or fast network activity. Conversely, interventions that widen Δ*V*_margin_ or reduce trigger efficacy would be expected to reduce preferential reactivation, attenuate vCA1 hyperactivity, improve synchrony-related readouts, and weaken downstream markers of network destabilization. The framework further predicts that combination strategies should outperform single-axis manipulations if the low-margin state is indeed maintained jointly by loss of excitability reserve, trigger structure, and feedback stabilization.

An important next step would be to integrate such single-cell electrophysiological measurements with *in vivo* network-level readouts, including reactivation probability, spike coupling to fast events, and limbic–cortical synchrony, in order to determine whether widening Δ*V*_margin_ causally reduces replay-prone network dynamics. At a more translational level, the same logic could later be extended to patient-derived cellular models or biomarker-oriented human studies designed to test whether low-margin states are associated with excessive hippocampal reactivity and whether multi-axis interventions produce stronger normalization than single-axis approaches.

These predictions are directly falsifiable. If supported empirically, the vCA1-centered framework developed here may also prove informative for other conditions characterized by excitability instability, particularly temporal lobe epilepsy and Alzheimer’s disease, in which analogous low-margin dynamics may emerge in regional nodes other than vCA1. In Alzheimer’s disease, this extension appears especially relevant because convergent work increasingly links early circuit dysfunction to regional hyperexcitability, excitation–inhibition imbalance, and ion-channel- and Ca^2+^-related mechanisms that could plausibly reduce effective excitability reserve. In this broader formulation, a central empirical question is not only whether Δ*V*_margin_ narrowing occurs, but where it emerges first and how it shapes phenotype-specific downstream network destabilization (Ciccone et al., 2019; Goodman et al., 2025; Haberman et al., 2017; Li et al., 2025; Targa Dias Anastacio et al., 2022).

## Conclusion

The present work proposes that the excitability margin (Δ*V*_margin_) may serve as a simple quantitative variable linking chronic stress, inflammatory influences, transient reactivation-related increases in excitability, and additional pro-excitatory modifiers to maladaptive circuit reactivation. Within the vCA1-centered model developed here, Δ*V*_margin_ may decrease from approximately 18.4 mV to ∼6.0 mV, and under additional “hits,” potentially to the range of 3–4 mV or even 1–2 mV. Because these values fall within the amplitude range of physiologically reported transient depolarizing events, the hypothesis of increased reactivation susceptibility appears biophysically plausible.

In this framework, narrowing of Δ*V*_margin_ is interpreted as a candidate excitability-based gating mechanism that may increase the probability of preferential reactivation of previously sensitized neuronal populations and thereby contribute to phenotype-specific circuit destabilization. Repeated reactivation may then promote replay-dependent plastic stabilization, while stress–inflammatory–redox–inhibitory feedback processes help maintain a chronically low-margin state.

The model is explicitly falsifiable. If valid, interventions that widen Δ*V*_margin_ or reduce trigger efficacy should reduce hyperreactivity in the vCA1/vHipp system analyzed here and weaken secondary processes that sustain pathological network states. In psychosis-like phenotypes, a first-order predicted effect would be attenuation of hippocampal hyperactivity and, secondarily, reduction of hippocampus-driven dopaminergic dysregulation.

If supported empirically, the present vCA1-centered framework may also prove informative for other conditions characterized by excitability instability, including temporal lobe epilepsy and Alzheimer’s disease. More broadly, the model is intended not only as an account of schizophrenia-, depression-, and trauma-related trajectories, but as a region-anchored formulation of a wider excitability-based destabilization logic in which phenotypes may diverge less by whether low-margin dynamics occur than by where they emerge first and which downstream feedback cascades subsequently stabilize them.

## Data Availability

The original contributions presented in the study are included in the article/Supplementary material, further inquiries can be directed to the corresponding author/s.
