# Supplementary material for: Excitability-margin narrowing as a candidate gating mechanism for maladaptive circuit reactivation: a ventral CA1-centered model
Source: Front Behav Neurosci. 2026 Apr 30;20:1839983. doi: 10.3389/fnbeh.2026.1839983 (PMC13171746; doi:10.3389/fnbeh.2026.1839983)
Supplement: Supplementary file 1 [file Data_Sheet_1.pdf]

Supplementary Discussion

Introduction to Phenotypic Trajectories

The following Supplementary Discussion sections present phenotype-specific, directionally literature-supported mechanistic sketches derived from the core ΔV_{margin} model. Their purpose is not to propose a complete, experimentally demonstrated causal chain, but rather to illustrate how a shared gating variable—a chronically narrowed excitability margin—may become embedded within distinct circuit architectures and dominant emotional contents.

In this sense, the following sections should be understood as biologically anchored extensions of the quantitative core, rather than as alternatives to it (1,2).

Within the proposed framework, a reduced ΔV_{margin} increases susceptibility of specific neuronal populations to preferential reactivation, while repeated cycles of reactivation may secondarily strengthen the same loops through synaptic plasticity, stress-related coupling, and network-level adaptations. From this shared starting point—common across phenotypes—fear-/salience-biased, rumination-biased, or trauma-reactivation–biased trajectories may emerge, depending on which circuits become preferentially stabilized and which emotional contents dominate the reactivated memory traces (1,2).

SD1. Fear-/Salience-Biased Loop Consistent with an SZ-like Phenotype

SD1.1. Circuit Architecture

If circuits related to fear processing and aberrant salience attribution become preferentially stabilized, a chronically narrowed excitability margin may promote a fear-/salience-biased trajectory consistent with SZ-like phenotypes.

This direction is aligned with literature indicating that schizophrenia is associated with abnormal recruitment of limbic structures—particularly the amygdala—during processing of both emotional and neutral stimuli. In addition, auditory hallucinations have been linked to increased θ–γ coupling within left temporo-frontal regions (3,4).

Within this framework, the broader hippocampal–amygdalar–prefrontal salience network represents a plausible circuit in which a reduced excitability margin may favor repeated reactivation of threat- or salience-tagged representations.

SD1.2. Neuromodulatory Trigger

In this trajectory, a single fear-memory recall or an additional stress episode may act as a trigger that transiently amplifies an already sensitized circuit.

The literature on stress and memory indicates that glucocorticoids and noradrenergic activation modulate both encoding quality and subsequent retrieval. Under stress, systems may shift toward less flexible, more habitual behavioral control modes (5–7).

Concurrently, physiological data demonstrate that ~4 Hz oscillations can coordinate activity across PFC, VTA, and hippocampus and are coupled to hippocampal theta rhythms, providing a plausible temporal framework for strengthening neuromodulatory drive within this loop (8).

In this sense, reactivation of a fear-related engram may not merely constitute passive retrieval but may transiently enhance plastic readiness across the entire circuit, increasing susceptibility to further reinforcement.

SD1.3. E/I Shift, Redox Load, and PV/PNN Vulnerability

If such reactivation cycles recur repeatedly, a gradual shift in excitation–inhibition balance may follow.

Magnetic resonance spectroscopy (MRS) studies and meta-analyses report heterogeneous but recurrent abnormalities in GABAergic and glutamatergic metabolites in medial frontal regions in schizophrenia. The direction and magnitude of effects depend on age, illness stage, and region examined, consistent with a model of local E/I vulnerability rather than a single global imbalance (9–13).

Post-mortem studies further indicate substantial reductions in perineuronal nets (PNNs) in the prefrontal cortex—on the order of ~70–76% in layers III and V—consistent with vulnerability of inhibitory microenvironments, including PV-interneuron–related circuits, to long-term destabilization (14).

Within this interpretation, repeated reactivation of fear-/salience-biased loops may not only transiently increase excitability but, over time, contribute to a state in which inhibitory control becomes less precise and less stable.

SD1.4. Oscillatory Output

If inhibitory precision and timing degrade, second-order effects would be expected at the level of network rhythms.

This is consistent with widely reported beta/gamma oscillatory disorganization and reduced long-range synchrony in schizophrenia, phenomena often attributed to dysfunction of GABAergic rhythm-generating interneuron networks (15).

In this context, the increased θ–γ coupling observed during auditory hallucinations may be interpreted as a state in which reactivation of specific content becomes more strongly entrained to locally privileged limbic–salience dynamics, while cortical regulatory control remains relatively weakened (4).

Functionally, this pattern is consistent with a shift toward network organization relatively more dominated by limbic–salience dynamics.

SD1.5. Structural and Plastic Stabilization

At later stages, repeated reactivation may become structurally stabilized.

Experimental work shows that chronic stress induces dendritic remodeling in medial prefrontal cortex, including approximately 20% retraction of apical dendritic arbors in pyramidal neurons, consistent with weakened top-down regulation of limbic circuits (16).

Fear conditioning promotes AMPA receptor insertion into lateral amygdala synapses, strengthening threat encoding. Epigenetic modifications associated with fear memory include changes in histone acetylation and DNA methylation at the BDNF locus in hippocampus, particularly at promoter IV/exon IV (17,18).

Additionally, somatostatin interneuron microcircuits in the dentate gyrus regulate engram ensemble size, providing a mechanism through which disrupted inhibition may increase cellular recruitment into memory traces (19).

Under this framework, the circuit becomes not only more reactive but also progressively more efficient at stabilizing its own preferential status.

SD1.6. Driving Sequence

Within the proposed model, the following directionally supported sequence can be outlined:

fear-/salience-engram reactivation

→ increased stress and glucocorticoid drive + strengthened phase coordination across the hippocampus–PFC–VTA axis

→ local E/I shift and weakened inhibitory control

→ PV/PNN destabilization and impaired beta/gamma synchrony

→ weakened cortical regulation with relative privileging of limbic processing

→ increased susceptibility to reactivation of the same content.

This sequence does not constitute proof of a complete closed causal chain within a single experiment, but is directionally consistent with literature describing stress modulation of memory, limbic biases in schizophrenia, oscillatory dysregulation, and vulnerability of PV/PNN-associated inhibitory systems (5,8,14,15).

SD2. Sadness-/Rumination-Biased Loop Consistent with an MDD-like Phenotype

SD2.1. Circuit Architecture

If circuits associated with negative valence, self-referential processing, and rumination become preferentially stabilized, a chronically narrowed excitability margin may promote a sadness-/rumination-biased trajectory consistent with MDD-like phenotypes.

This direction aligns with literature indicating that depression is associated with increased functional coupling between the subgenual prefrontal cortex (sgPFC/sgACC) and the default mode network (DMN), with the strength of this connectivity often correlating with rumination severity (20). In practical terms, the sgACC–DMN axis, embedded within a broader prefrontal–limbic circuitry, represents a plausible candidate system in which a reduced ΔV_{margin} may increase the probability of repeated reactivation of negatively valenced activity patterns.

SD2.2. Neuromodulatory Trigger

Within this trajectory, recurrent stress episodes or reactivation of negatively valenced memory traces could function as triggers that reinforce an already sensitized circuit.

This interpretation is consistent with classical findings demonstrating elevated CRF-like immunoreactivity in the cerebrospinal fluid of patients with major depression, supporting the hypothesis of chronic CRF/HPA-axis dysregulation [21]. In parallel, cerebrospinal fluid monoamine metabolite profiles in depression indicate reduced levels of homovanillic acid (HVA), without comparably consistent effects for 5-HIAA or MHPG, suggesting the possibility of relative dopaminergic attenuation in at least a subset of depressive phenotypes [22].

Within this framework, repeated reactivation may co-occur with a neuroendocrine and monoaminergic milieu that favors stabilization of negative cognitive bias.

SD2.3. E/I Shift in Prefrontal–Limbic Nodes

If such reactivation cycles recur, a gradual shift in excitation–inhibition balance within prefrontal–limbic nodes may follow.

Integrative reviews combining clinical, post-mortem, and preclinical data indicate that depression is associated with deficits in GABAergic transmission in the prefrontal cortex,

alongside accompanying glutamatergic abnormalities. This pattern is consistent with a model of local E/I destabilization rather than a uniform global imbalance (21,22).

In practical terms, the circuit responsible for self-referential negative processing may not only become more frequently reactivated but may also gradually lose inhibitory precision, thereby facilitating continued dominance of the same activity patterns.

SD2.4. PNN/PV Vulnerability and Network Control

A further level of destabilization may involve weakening of structural components that support precise inhibitory regulation.

Experimental data indicate that in the chronic unpredictable mild stress (CUMS) model, perineuronal net (PNN) density and aggrecan expression decrease in the prelimbic cortex, changes that co-occur with depression-like phenotypes (23). This is consistent with broader literature indicating that PNNs exert protective effects on fast-spiking, PV-associated interneuron circuits under oxidative stress conditions, and that their weakening may reduce inhibitory stability (24).

Under this interpretation, chronic reactivation of ruminative loops may progressively lead to a state in which inhibitory control becomes less effective and the network more readily stabilizes its own preferential configuration.

SD2.5. Oscillatory Output and Rumination

If inhibitory control and state-switching flexibility weaken, second-order consequences would be expected at the level of oscillatory and functional organization.

This interpretation is consistent with findings that rumination in depression is associated with increased sgPFC/sgACC coupling to the DMN, and that relative dominance of the DMN over the task-positive network (TPN) correlates with more maladaptive forms of rumination (20,25).

EEG studies involving memory-related tasks further suggest that ruminative and anxious traits may co-occur with altered alpha/beta dynamics, consistent with difficulty disengaging attentional resources from negatively valenced internal content (26).

Within this context, a chronically reduced excitability margin would not necessarily generate a novel type of neural activity, but may increase the probability that the network repeatedly enters states in which the same ruminative contents are reactivated and sustained.

SD2.6. Structural and Synaptic Stabilization

At later stages, repeated reactivation may become structurally consolidated.

Post-mortem data indicate reduced expression of synapse-related genes and decreased synapse number in the dorsolateral prefrontal cortex of patients with MDD (27). Chronic stress models further demonstrate gray matter reductions in ACC and hippocampus, alongside dendritic spine density losses reaching up to approximately 60% on selected dendritic segments (28).

Taken together, these findings depict a scenario in which the ruminative circuit is not only repeatedly reactivated but progressively loses structural flexibility and capacity to revert to a more adaptive network configuration.

SD2.7. Driving Sequence

Within the proposed framework, the following directionally supported sequence may be outlined:

reactivation of negatively valenced / ruminative engram

→ chronic co-occurrence of CRF/HPA bias and possible relative dopaminergic attenuation

→ enhanced sgACC–DMN coupling and impaired disengagement from self-referential state

→ local E/I shift in prefrontal–limbic nodes

→ weakening of PNN/PV-associated inhibitory stabilization mechanisms

→ further stabilization of ruminative activity patterns

→ subsequent cycles of reactivation.

This outline does not constitute proof of a complete causal chain within a single experiment, but is directionally consistent with literature describing rumination, CRF/HPA dysregulation, GABAergic and glutamatergic alterations, and chronic synaptic–structural changes in depression.

SD3. Trauma-Reactivation–Biased Loop Consistent with a PTSD-like Phenotype

SD3.1. Circuit Architecture and Neuromodulatory Trigger

If circuits associated with trauma and aversive stimuli become preferentially stabilized, a chronically narrowed excitability margin may promote a trauma-reactivation–biased trajectory consistent with PTSD-like phenotypes.

This direction aligns with literature indicating that trauma recall and exposure to trauma-related cues strongly engage limbic and stress-related systems. A plausible amplification mechanism involves the locus coeruleus (LC) → basolateral amygdala (BLA) projection; experimental studies demonstrate that activation of this pathway increases norepinephrine release within the amygdala and enhances anxiety- and aversion-related behaviors (29,30).

In parallel, elevated cerebrospinal fluid CRF levels have been reported in patients with PTSD, consistent with a model of chronic stress-system hyperactivation (31).

Within this framework, a single trauma reminder would not merely retrieve a stored trace, but could transiently amplify an already sensitized circuit through the combined influence of noradrenergic drive and CRF-dependent stress signaling.

SD3.2. E/I Shift in Selected Cortico-Limbic Nodes

If such reactivation occurs repeatedly, a natural next step would be a shift in excitation–inhibition balance within selected cortico-limbic nodes.

Magnetic resonance spectroscopy (MRS) data indicate reduced GABA levels in the right anterior insula (approximately 30% lower GABA/Cr) and elevated glutamate levels in the right hippocampus in PTSD, consistent with localized vulnerability toward increased excitability (32,33).

In the context of the main model proposed in this work, this pattern is particularly relevant, as it aligns with a state in which a reduced $\Delta V_{\mathrm{margin}}$ increases the likelihood of repeated reactivation specifically within trauma-related circuits.

SD3.3. PNN/PV Vulnerability, Oscillations, and Weakened Cortical Control

If the E/I shift persists over time, further destabilization may involve degradation of inhibitory precision and network synchrony.

Broader stress literature indicates that chronic stress exposure may alter PV-interneuron phenotype, while perineuronal nets (PNNs) serve protective functions for fast-spiking PV-associated interneuron circuits, supporting the stability of precise inhibition (24,34).

PTSD has also been associated with abnormalities in oscillatory activity and network synchrony. Neuroimaging studies consistently support a pattern of relatively reduced vmPFC activity during trauma recall, response inhibition tasks, and extinction-related processes, alongside disrupted network organization (29,35,36).

Within this framework, a chronically reactivated trauma-related circuit may not only become more frequently engaged but may progressively lose cortical gating mechanisms required for suppression or extinction of intrusive activity patterns.

SD3.4. Synaptic and Memory Stabilization

At later stages, repeated reactivation may become consolidated through plastic changes within the hippocampus–amygdala axis.

Experimental studies demonstrate that aversive learning enhances synaptic plasticity within the amygdala. In particular, activity of the vCA1 → basolateral amygdala (BA) projection paired with aversive stimuli contributes to contextual fear memory encoding, and conditioning leads to selective strengthening of subsets of vCA1–BA synapses (37).

This finding aligns well with a model in which a reduced excitability margin not only facilitates reactivation but also progressively promotes more durable privileging of the same trauma-biased memory pathway.

SD3.5. Driving Sequence

Within the proposed framework, the following directionally supported sequence may be outlined:

trauma reminder / reactivation of aversive engram

→ increased LC→BLA drive and CRF-dependent stress signaling

→ local E/I shift in selected cortico-limbic nodes

→ impaired inhibitory control and synchrony (including weakened vmPFC gating)

→ increased susceptibility to intrusive reactivation of the same content

→ repeated reactivation cycles and progressive trace stabilization.

This outline does not constitute proof of a complete closed causal chain within a single experiment, but remains directionally consistent with literature on noradrenergic drive, CRF signaling, E/I imbalance, vmPFC dysfunction, and hippocampus–amygdala plasticity in PTSD-like states.

SD4. Memory Co-Allocation, Convergence onto "Hot Spots," and the Possibility of Phenotypic Mixing

SD4.1. The "Capture" Mechanism: Why New Traces May Be Allocated to Already Privileged Cells

One of the most natural extensions of the ΔV_{margin} model is the assumption that neurons with a chronically reduced excitability margin may not only be more easily reactivated, but may also have an increased probability of capturing newly encoded memory traces.

This interpretation is consistent with allocation literature showing that neurons with higher intrinsic excitability are preferentially recruited into engrams, and that transient increases in excitability following one event can bias representation of a subsequent event toward the same cellular ensemble (38,39).

Within this framework, a reduced ΔV_{margin} would not only increase the probability of reactivating an existing trace, but could also increase the likelihood that new experiences become at least partially encoded within the same already privileged neuronal population.

SD4.2. Linking Temporally Proximal Events and Engram Overlap

The mechanism is particularly consistent with studies of memory linking across temporally proximal events. Two distinct experiences acquired within a short temporal window can be represented by partially overlapping CA1 ensembles, and recall of one can increase the probability of recalling the other; this effect has been interpreted as arising from transient post-encoding increases in excitability that bias allocation of the second trace toward the same neuronal ensemble [42]. Similarly, interactions between engrams appear to be regulated by neuronal excitability and competitive allocation processes [41]. In the context of the present model, these findings suggest that a chronically reduced excitability margin may prolong or amplify a “capture-ready” state, enabling a previously reactive circuit to more readily attract additional traces encoded in close temporal succession—or potentially experiences that are partially functionally or affectively related [41,42].

SD4.3. Convergence onto Local High-Excitability “Hot Spots”

If such a process repeats over time, a natural consequence would be progressive convergence of multiple memory traces onto a local “hot spot”—a relatively small ensemble of neurons characterized by elevated excitability, strengthened connectivity, and increased probability of future recruitment.

In the case of aversive content, the vCA1 → basolateral amygdala (BA) pathway represents a particularly plausible candidate node. Contextual fear conditioning selectively strengthens subsets of vCA1→BA synapses, and activity within this projection contributes directly to contextual fear memory encoding (37).

Thus, once privileged, an ensemble within such a circuit may increasingly function as an attractor for subsequent emotionally congruent traces.

SD4.4. ECM/PNN-Mediated Stabilization of the “Hot Spot”

An additional stabilizing layer may involve extracellular matrix (ECM) components and perineuronal nets (PNNs).

Extracellular matrix (ECM) and perineuronal net (PNN) structures not only regulate plasticity but also participate in memory control and memory-related pathology [43]. In practical terms, once a neuronal ensemble becomes a repeatedly reactivated “hot spot,” its relative stabilization may be supported not only by synaptic strengthening but also by the surrounding extracellular matrix environment, which may help maintain connection architecture and limit return to a more flexible network configuration [43].

In this sense, long-term stabilization of a “hot spot” may reflect the combined effects of altered excitability, synaptic plasticity, and ECM/PNN-imposed structural constraints.

SD4.5. A Potential Mechanism for Phenotypic Mixing and Trajectory Transitions

Within the proposed framework, such processes may naturally give rise to phenotypic mixing.

If a dominant, already privileged circuit begins capturing traces of partially different emotional content, one trajectory may not fully replace another but may begin to overlap with it. For example, a circuit initially sadness-/rumination-biased may progressively capture fear-/salience-related content. Conversely, a chronically trauma-biased circuit may begin to bias new experiences toward heightened salience attribution, intrusiveness, or negative self-referential processing.

In this interpretation, transitions such as MDD-like → SZ-like, PTSD-like → salience-biased, or mixed states may reflect gradual expansion of the range of content being integrated into the same privileged network core, rather than entirely distinct disease processes.

This logic is consistent with mechanisms of co-allocation and memory linking, but in the present work remains a mechanistic hypothesis rather than a clinically demonstrated causal chain.

SD4.6. Driving Sequence

Within the proposed framework, the following directionally supported sequence may be outlined:

chronically reduced $\Delta V_{\mathrm{margin}}$

→ preferential reactivation of a privileged ensemble

→ increased relative excitability and strengthened connectivity within that ensemble

→ biased allocation of subsequent events to the same cellular population

→ partial engram overlap for temporally proximal or affectively related experiences

→ formation of an increasingly strong, emotionally polarized “hot spot”

→ potential overlap of contents and transitions between phenotypic trajectories.

This outline is consistent with literature on allocation, linking, and fear-circuit plasticity, but does not constitute proof of a complete, closed causal chain for clinical transitions between disorders.

SD4.7. Regional Ignition Sites and a Partially Shared Low-Margin Logic Across Phenotypes

A broader implication of the present framework is that the critical event may not be the identity of one privileged anatomical structure, but the emergence of a chronically reduced excitability margin within a phenotype-relevant circuit node. In the current manuscript, ventral CA1 is used as the principal model node because it provides a stress-sensitive and affectively relevant entry point for quantitative analysis. However, the same general logic does not require that low-margin dynamics originate uniquely in vCA1.

More generally, different phenotypes may involve partially shared excitability-based destabilization emerging first in different regional “ignition sites.” In some conditions, the most vulnerable node may lie within ventral hippocampal–amygdalar circuitry; in others, it may arise in dorsal hippocampal or entorhinal networks, fronto-limbic control systems, cortico-

striatal loops, or other distributed nodes depending on the dominant risk architecture and computational role of the affected circuit. In this sense, phenotypes may diverge less by whether ΔV_{margin} narrowing occurs than by where it emerges first and which neuronal ensembles become preferentially reactivated and progressively stabilized thereafter.

Within this broader interpretation, heterogeneous upstream pressures—including chronic stress, inflammatory signaling, redox burden, metabolic stress, neuromodulatory dysregulation, and other excitability-destabilizing influences—would converge on the same biophysical consequence: narrowing of the remaining distance between resting membrane potential and spike threshold. Once a local circuit enters such a low-margin state, ordinary physiological events become more likely to contribute to threshold crossing, preferential reactivation, and repeated recruitment of the same neuronal populations. Recurrent reactivation may then promote replay-dependent synaptic strengthening, reduced inhibitory precision, oscillatory destabilization, and secondary feedback processes that help maintain the circuit in a chronically vulnerable state.

Under this framework, downstream divergence across phenotypes would be shaped primarily by the anatomical location and computational role of the affected node. A low-margin state arising in hippocampal–amygdalar circuits may preferentially bias fear-, salience-, or trauma-related trajectories; in fronto-limbic self-referential networks, it may favor ruminative or negative-valence dynamics; in other conditions, analogous low-margin dynamics may plausibly contribute to seizure-prone or neurodegeneration-linked patterns, depending on which ensembles are repeatedly captured and reinforced. Thus, the proposed model points to a partially shared instability logic across phenotypes, while preserving the importance of regional specificity in determining outward clinical expression.

In this view, the common backbone is not a single disease-specific pathway, but a sequence of directionally related events:

heterogeneous risk factors / chronic insults

→ regional excitability-margin narrowing in a phenotype-relevant ignition site

→ threshold-near network regime

→ preferential reactivation of vulnerable ensembles

→ replay-dependent strengthening and impaired inhibitory/synchrony control

→ feedback stabilization of a chronically low-margin state

→ phenotype-shaped downstream circuit dysfunction.

This formulation remains a mechanistic hypothesis rather than an experimentally demonstrated transdiagnostic causal chain. Its value lies in providing a common biophysical language through which distinct phenotypes may be interpreted as regionally differentiated expressions of partially shared low-margin dynamics.

References

1. Josselyn SA, Tonegawa S. Memory engrams: Recalling the past and imagining the future. Science. 2020 Jan 3;367(6473):eaaw4325. doi:10.1126/science.aaw4325

2. Rao-Ruiz P, Visser E, Mitrić M, Smit AB, Van Den Oever MC. A Synaptic Framework for the Persistence of Memory Engrams. Front Synaptic Neurosci. 2021 Mar 24;13:661476. doi:10.3389/fnsyn.2021.661476

3. Hall J, Whalley HC, McKirdy JW, Romaniuk L, McGonigle D, McIntosh AM, et al. Overactivation of Fear Systems to Neutral Faces in Schizophrenia. Biol Psychiatry. 2008 Jul;64(1):70–3. doi:10.1016/j.biopsych.2007.12.014

4. Koutsoukos E, Angelopoulos E, Maillis A, Papadimitriou GN, Stefanis C. Indication of increased phase coupling between theta and gamma EEG rhythms associated with the experience of auditory verbal hallucinations. Neurosci Lett. 2013 Feb;534:242–5. doi:10.1016/j.neulet.2012.12.005

5. Schwabe L, Wolf OT, Oitzl MS. Memory formation under stress: Quantity and quality. Neurosci Biobehav Rev. 2010 Mar;34(4):584–91. doi:10.1016/j.neubiorev.2009.11.015

6. Schwabe L, Wolf OT. Stress-induced modulation of instrumental behavior: From goal-directed to habitual control of action. Behav Brain Res. 2011 Jun;219(2):321–8. doi:10.1016/j.bbr.2010.12.038

7. Schönfeld P, Ackermann K, Schwabe L. Remembering under stress: Different roles of autonomic arousal and glucocorticoids in memory retrieval. Psychoneuroendocrinology. 2014 Jan;39:249–56. doi:10.1016/j.psyneuen.2013.09.020

8. Fujisawa S, Buzsáki G. A 4 Hz Oscillation Adaptively Synchronizes Prefrontal, VTA, and Hippocampal Activities. Neuron. 2011 Oct;72(1):153–65. doi:10.1016/j.neuron.2011.08.018

9. Marsman A, Van Den Heuvel MP, Klomp DWJ, Kahn RS, Luijten PR, Hulshoff Pol HE. Glutamate in Schizophrenia: A Focused Review and Meta-Analysis of 1H-MRS Studies. Schizophr Bull. 2013 Jan;39(1):120–9. doi:10.1093/schbul/sbr069

10. Rowland LM, Kontson K, West J, Edden RA, Zhu H, Wijtenburg SA, et al. In Vivo Measurements of Glutamate, GABA, and NAAG in Schizophrenia. Schizophr Bull. 2013 Sep;39(5):1096–104. doi:10.1093/schbul/sbs092

11. Rowland LM, Krause BW, Wijtenburg SA, McMahon RP, Chiappelli J, Nugent KL, et al. Medial frontal GABA is lower in older schizophrenia: a MEGA-PRESS with macromolecule suppression study. Mol Psychiatry. 2016 Feb;21(2):198–204. doi:10.1038/mp.2015.34

12. Egerton A, Modinos G, Ferrera D, McGuire P. Neuroimaging studies of GABA in schizophrenia: a systematic review with meta-analysis. Transl Psychiatry. 2017 Jun 6;7(6):e1147–e1147. doi:10.1038/tp.2017.124

13. Simmonite M, Steeby CJ, Taylor SF. Medial Frontal Cortex GABA Concentrations in Psychosis Spectrum and Mood Disorders: A Meta-analysis of Proton Magnetic Resonance Spectroscopy Studies. Biol Psychiatry. 2023 Jan;93(2):125–36. doi:10.1016/j.biopsych.2022.08.004

14. Mauney SA, Athanas KM, Pantazopoulos H, Shaskan N, Passeri E, Berretta S, et al. Developmental Pattern of Perineuronal Nets in the Human Prefrontal Cortex and Their Deficit in Schizophrenia. Biol Psychiatry. 2013 Sep;74(6):427–35. doi:10.1016/j.biopsych.2013.05.007

15. Uhlhaas PJ, Singer W. Abnormal neural oscillations and synchrony in schizophrenia. Nat Rev Neurosci. 2010 Feb;11(2):100–13. doi:10.1038/nrn2774

16. Radley JJ, Sisti HM, Hao J, Rocher AB, McCall T, Hof PR, et al. Chronic behavioral stress induces apical dendritic reorganization in pyramidal neurons of the medial prefrontal cortex. Neuroscience. 2004 Jan;125(1):1–6. doi:10.1016/j.neuroscience.2004.01.006

17. Rumpel S, LeDoux J, Zador A, Malinow R. Postsynaptic Receptor Trafficking Underlying a Form of Associative Learning. Science. 2005 Apr;308(5718):83–8. doi:10.1126/science.1103944

18. Lubin FD, Roth TL, Sweatt JD. Epigenetic Regulation of *bdnf* Gene Transcription in the Consolidation of Fear Memory. J Neurosci. 2008 Oct 15;28(42):10576–86. doi:10.1523/JNEUROSCI.1786-08.2008

19. Stefanelli T, Bertollini C, Lüscher C, Muller D, Mendez P. Hippocampal Somatostatin Interneurons Control the Size of Neuronal Memory Ensembles. Neuron. 2016 Mar;89(5):1074–85. doi:10.1016/j.neuron.2016.01.024

20. Hamilton JP, Farmer M, Fogelman P, Gotlib IH. Depressive Rumination, the Default-Mode Network, and the Dark Matter of Clinical Neuroscience. Biol Psychiatry. 2015 Aug;78(4):224–30. doi:10.1016/j.biopsych.2015.02.020

21. Fogaça MV, Duman RS. Cortical GABAergic Dysfunction in Stress and Depression: New Insights for Therapeutic Interventions. Front Cell Neurosci. 2019 Mar 12;13:87. doi:10.3389/fncel.2019.00087

22. Duman RS, Sanacora G, Krystal JH. Altered Connectivity in Depression: GABA and Glutamate Neurotransmitter Deficits and Reversal by Novel Treatments. Neuron. 2019 Apr;102(1):75–90. doi:10.1016/j.neuron.2019.03.013

23. Yu Z, Chen N, Hu D, Chen W, Yuan Y, Meng S, et al. Decreased Density of Perineuronal Net in Prelimbic Cortex Is Linked to Depressive-Like Behavior in Young-Aged Rats. Front Mol Neurosci. 2020 Jan 28;13:4. doi:10.3389/fnmol.2020.00004

24. Cabungcal JH, Steullet P, Morishita H, Kraftsik R, Cuenod M, Hensch TK, et al. Perineuronal nets protect fast-spiking interneurons against oxidative stress. Proc Natl Acad Sci. 2013 May 28;110(22):9130–5. doi:10.1073/pnas.1300454110

25. Hamilton JP, Furman DJ, Chang C, Thomason ME, Dennis E, Gotlib IH. Default-Mode and Task-Positive Network Activity in Major Depressive Disorder: Implications for Adaptive and Maladaptive Rumination. Biol Psychiatry. 2011 Aug;70(4):327–33. doi:10.1016/j.biopsych.2011.02.003

26. Forner-Phillips NA, Mills C, Ross RS. Tendency to ruminate and anxiety are associated with altered alpha and beta oscillatory power dynamics during memory for contextual details. Cogn Affect Behav Neurosci. 2020 Aug;20(4):698–716. doi:10.3758/s13415-020-00797-2

27. Kang HJ, Voleti B, Hajszan T, Rajkowska G, Stockmeier CA, Licznerski P, et al. Decreased expression of synapse-related genes and loss of synapses in major depressive disorder. Nat Med. 2012 Sep;18(9):1413–7. doi:10.1038/nm.2886

28. Kassem MS, Lagopoulos J, Stait-Gardner T, Price WS, Chohan TW, Arnold JC, et al. Stress-Induced Grey Matter Loss Determined by MRI Is Primarily Due to Loss of Dendrites and Their Synapses. Mol Neurobiol. 2013 Apr;47(2):645–61. doi:10.1007/s12035-012-8365-7

29. Hughes KC, Shin LM. Functional neuroimaging studies of post-traumatic stress disorder. Expert Rev Neurother. 2011 Feb;11(2):275–85. doi:10.1586/ern.10.198

30. McCall JG, Siuda ER, Bhatti DL, Lawson LA, McElligott ZA, Stuber GD, et al. Locus coeruleus to basolateral amygdala noradrenergic projections promote anxiety-like behavior. eLife. 2017 Jul 14;6:e18247. doi:10.7554/eLife.18247

31. Bremner JD, Licinio J, Darnell A, Krystal JH, Owens MJ, Southwick SM, et al. Elevated CSF corticotropin-releasing factor concentrations in posttraumatic stress disorder. Am J Psychiatry. 1997 May;154(5):624–9. doi:10.1176/ajp.154.5.624 PubMed PMID: 9137116; PubMed Central PMCID: PMC3233756.

32. Rosso IM, Weiner MR, Crowley DJ, Silveri MM, Rauch SL, Jensen JE. INSULA AND ANTERIOR CINGULATE GABA LEVELS IN POSTTRAUMATIC STRESS DISORDER: PRELIMINARY FINDINGS USING MAGNETIC RESONANCE SPECTROSCOPY: Research Article: Insula GABA in PTSD. Depress Anxiety. 2014 Feb;31(2):115–23. doi:10.1002/da.22155

33. Rosso IM, Crowley DJ, Silveri MM, Rauch SL, Jensen JE. Hippocampus Glutamate and N-Acetyl Aspartate Markers of Excitotoxic Neuronal Compromise in Posttraumatic Stress Disorder. Neuropsychopharmacology. 2017 Jul;42(8):1698–705. doi:10.1038/npp.2017.32

34. Perlman G, Tanti A, Mechawar N. Parvalbumin interneuron alterations in stress-related mood disorders: A systematic review. Neurobiol Stress. 2021 Nov;15:100380. doi:10.1016/j.ynstr.2021.100380

35. Jovanovic T, Ely T, Fani N, Glover EM, Gutman D, Tone EB, et al. Reduced neural activation during an inhibition task is associated with impaired fear inhibition in a traumatized civilian sample. Cortex. 2013 Jul;49(7):1884–91. doi:10.1016/j.cortex.2012.08.011

36. Dunkley BT, Doesburg SM, Jetly R, Sedge PA, Pang EW, Taylor MJ. Characterising intra- and inter-intrinsic network synchrony in combat-related post-traumatic stress disorder. Psychiatry Res Neuroimaging. 2015 Nov;234(2):172–81. doi:10.1016/j.pscychresns.2015.09.002

37. Kim WB, Cho JH. Encoding of contextual fear memory in hippocampal–amygdala circuit. Nat Commun. 2020 Mar 13;11(1):1382. doi:10.1038/s41467-020-15121-2

38. Josselyn SA, Frankland PW. Memory Allocation: Mechanisms and Function. Annu Rev Neurosci. 2018 Jul 8;41(1):389–413. doi:10.1146/annurev-neuro-080317-061956

39. Rashid AJ, Yan C, Mercaldo V, Hsiang HL (Liz), Park S, Cole CJ, et al. Competition between engrams influences fear memory formation and recall. Science. 2016 Jul 22;353(6297):383–7. doi:10.1126/science.aaf0594
