# Supplementary material for: Excitability-margin narrowing as a candidate gating mechanism for maladaptive circuit reactivation: a ventral CA1-centered model
Source: Front Behav Neurosci. 2026 Apr 30;20:1839983. doi: 10.3389/fnbeh.2026.1839983 (PMC13171746; doi:10.3389/fnbeh.2026.1839983)
Supplement: Supplementary file 2 [file Supplementary_file_1.pdf]

Supplementary Methods 1

S1.1. Definition of the Excitability Margin and Stress Module (CRS) — Baseline Margin

The baseline excitability margin was defined as:

$$\Delta V_{\text{margin},0} = V_{\text{thr},0} - V_{\text{rest},0}$$

This parameter describes the voltage distance between resting membrane potential and spike threshold under baseline conditions. In the present model, $\Delta V_{\text{margin},0}$ was treated as a static reference point against which subsequent narrowing of the functional excitability reserve was evaluated.

S1.2. Effective Margin During Synaptic Activity

During network activity, the effectiveness of $GABA_{\text{A}}$ receptor–mediated inhibition depends not only on receptor conductance but also on the chloride gradient and the reversal potential for GABAergic currents (E_{GABA}). A depolarizing shift of E_{GABA} weakens the functional efficacy of chloride-dependent inhibition, reducing the ability of GABAergic signaling to maintain membrane potential at a distance from spike threshold (1).

To capture this effect in a simplified manner, an inhibitory term ΔV_{inh} was introduced while preserving sign convention: more negative values correspond to stronger inhibition, whereas a depolarizing shift of E_{GABA} shifts ΔV_{inh} toward zero.

The effective excitability margin was defined as:

$$\Delta V_{\text{eff}} = V_{\text{thr}} - (V_{\text{rest}} + \Delta V_{\text{inh}})$$

In this formulation, ΔV_{eff} represents the functional reserve to spike threshold under conditions of active inhibition.

S1.3. Empirical Data for Chronic Restraint Stress (CRS)

Using gramicidin-perforated patch recordings in CA1 pyramidal neurons, (2) demonstrated that chronic restraint stress (CRS) induces a significant depolarizing shift in the $GABA_{\text{A}}$ reversal potential:

$$E_{\text{GABA}}: -68.33 \pm 2.31 \text{ mV} \rightarrow -60.26 \pm 1.83 \text{ mV}$$

corresponding to:

$$\Delta E_{\text{GABA}} \approx +8.1 \text{ mV}$$

In the same study, no significant change in resting membrane potential was observed; however, a reduction in the minimal current required to reach action potential threshold (iAP, treated here as an operational equivalent of rheobase) was reported:

$$58.68 \pm 7.73 \text{ pA} \rightarrow 32.93 \pm 9.00 \text{ pA}$$

corresponding to a decrease to approximately 0.56 of control values. The authors also reported reduced phosphorylation of KCC2 at Ser940, consistent with impaired transporter function (2).

Collectively, these findings indicate that CRS is associated with both weakened chloride regulation and increased CA1 neuronal excitability.

S1.4. Working Parameter of the Stress Module

In the present model, stress-induced excitability enhancement following CRS was parameterized using a dimensionless coefficient based on the ratio of rheobase (operationally: iAP) values in stressed versus control groups:

$$\gamma_{\text{rh}} = \frac{I_{\text{rh,CRS}}}{I_{\text{rh,ctrl}}}$$

Based on (2):

$$\gamma_{\text{rh}} = \frac{32.93}{58.68} \approx 0.56$$

The coefficient γ_{rh} was treated as the primary numerical parameter describing stress-induced excitability increase in subsequent calculations. In the present framework, γ_{rh} should be interpreted as a phenomenological scaling term and operational index of functional compression of excitability reserve, rather than as a literal voltage-domain transformation.

S1.5. Mechanistic Status of the Chloride Effect

The reported shift in E_{GABA} and impairment of KCC2 function were treated in the present framework as mechanistic background of the CRS phenotype. However, this chloride-related effect was not inserted separately into the final calculations of the main scenario. Instead, it was assumed that the operational numerical description of CRS effects on the excitability margin is sufficiently captured by γ_{rh}, thereby avoiding double-counting of two parameters describing the same stress phenotype.

S1.6. Extrapolation from CA1 to vCA1

Direct gramicidin-perforated patch measurements following chronic restraint stress (CRS) are currently not available for ventral CA1 (vCA1). For this reason, the CA1 stress phenotype previously described in the literature was used as a model approximation for vCA1 (2).

This assumption is conservative. Ventral CA1 pyramidal neurons have been shown to be intrinsically more excitable than dorsal CA1 neurons: they fire in response to smaller current injections, exhibit a more depolarized resting membrane potential, and show higher input resistance (3). In this context, using general CA1 data as a starting point for vCA1 modeling should not lead to an obvious overestimation of activation susceptibility (3).

Supplementary Methods 2

Stress-Associated Inflammatory Component

S2.1. Acute Inflammatory Component as Reference Point

In an LPS-induced inflammation model, CA1 pyramidal neurons exhibit increased excitability, elevated input resistance, and significant depolarization of the resting membrane potential, without a significant change in action potential threshold, as reported by (4).

Based on approximate extraction from Fig. 2D of that study, LPS was assumed to depolarize the resting membrane potential of CA1 neurons by approximately:

$$\Delta V_{\text{rest}}^{\text{LPS}} \approx +6.0 \text{ mV}$$

This value was treated as a reference point for a strong acute inflammatory component.

S2.2. Rationale for an Inflammatory Component in Chronic Stress

Chronic stress is associated with neuroinflammatory activation within the hippocampus. Psychosocial stressors increase microglial activation in the hippocampus, and chronic restraint stress elevates IL-1β and IL-6 levels in hippocampal CA3 (5,6). These findings justify inclusion of a pro-inflammatory component as an additional model module accompanying stress (5,6).

Because the available literature does not provide a direct quantitative conversion factor between the magnitude of inflammation in CRS and in the LPS model, the stress-associated inflammatory component was treated as a conservative modeling assumption.

S2.3. Adopted Inflammatory Component in the Main Scenario

In the model, chronic stress was assumed to recruit approximately one-third of the depolarizing effect observed in the strong LPS model:

$$\Delta V_{\text{rest,infl,stress}} = \alpha_{\text{infl}} \cdot \Delta V_{\text{rest}}^{\text{LPS}}$$

where:

$$\alpha_{\text{infl}} = 0.33$$

Substituting values:

$$\Delta V_{\text{rest,infl,stress}} = 0.33 \times 6.0 \text{ mV} = 1.98 \text{ mV}$$

which was rounded to:

$$\Delta V_{\text{rest,infl,stress}} \approx +2.0 \text{ mV}$$

S2.4. Implementation in Calculations

Given that no significant change in spike threshold was observed under inflammatory conditions, the inflammatory module was implemented as a direct reduction of the difference $V_{thr} - V_{rest}$ by the same magnitude (4).

Accordingly, the following value was used in subsequent calculations for the main scenario:

$$\Delta V_{\text{rest,infl,stress}} = +2.0 \text{ mV}$$

Supplementary Methods 3

"Hot Spot": Transient Margin Narrowing During Engram Reactivation

S3.1. Rationale for the Module

Memory reactivation transiently increases the excitability of engram cells and facilitates subsequent retrieval of the memory trace (7). In addition, ventral CA1 (vCA1) neuronal ensembles participate in fear memory retrieval, and recall is associated with increased correlated activity among neurons previously involved in encoding the aversive stimulus (8).

On this basis, a transient excitability "hot spot" module was introduced to represent increased reactivation susceptibility within previously recruited neuronal populations.

S3.2. Numerical Parameter of the "Hot Spot" Module

The numerical parameter of this module was defined as a transient reduction in excitability margin:

$$\Delta V_{\text{hot}} = +3.2 \text{ mV}$$

This value was derived from data reported by (9), who showed that elevated CREB activity does not alter resting membrane potential but lowers action potential threshold to −38.8 ± 0.9 mV, compared with control groups (−35.4 ± 1.0 to −35.7 ± 1.0 mV).

Because V_{rest} remains unchanged, this corresponds to a direct reduction of the difference $V_{\text{thr}} - V_{\text{rest}}$ by approximately 3.1–3.4 mV. In the present model, this value was rounded to:

$$\Delta V_{\text{hot}} = 3.2 \text{ mV}$$

S3.3. Modeling Status of the Parameter

Because the referenced measurements were obtained from neurons of the lateral amygdala rather than ventral CA1 (vCA1), the value of ΔV_hot was treated as a phenomenological approximation of transient threshold lowering during engram reactivation, rather than as a region-specific direct measurement for vCA1 (9).

S3.4. Implementation in Calculations

The effect of engram reactivation was implemented as a direct subtraction of ΔV_{hot} from the effective margin after incorporation of prior model components:

$$\Delta V_{\text{eff,hot}} = \Delta V_{\text{eff,prev}} - \Delta V_{\text{hot}}$$

where $\Delta V_{\text{eff,prev}}$ denotes the effective margin after inclusion of previous model modules.

For subsequent calculations in the main scenario, the following value was used:

$$\Delta V_{\text{hot}} = +3.2 \text{ mV}$$

Supplementary Methods 4

Cumulative Narrowing of the Excitability Margin in the Main Scenario

S4.1. Baseline Margin for vCA1 Pyramidal Neurons

As the starting point for the main scenario, a static baseline excitability margin for vCA1 pyramidal neurons was defined as:

$$\Delta V_{\text{margin,0}} = V_{\text{thr,0}} - V_{\text{rest,0}}$$

For ventral CA1 pyramidal cells, the reference values are:

$$V_{\text{rest,0}} = -68.6 \text{ mV}$$
$$V_{\text{thr,0}} = -50.2 \text{ mV}$$

yielding:

$$\Delta V_{\text{margin,0}} = (-50.2) - (-68.6) = 18.4 \text{ mV}$$

These values were obtained from Table S4 of (10).

S4.2. Components of the Main Scenario

The main scenario incorporated three interacting modules:

1. Stress-induced excitability increase following CRS, described by the coefficient:

$$\gamma_{\text{rh}} = 0.56$$

2. An additional stress-associated inflammatory component:

$$\Delta V_{\text{rest,infl,stress}} = +2.0 \text{ mV}$$

3. A transient engram-reactivation “hot spot” module:

$$\Delta V_{\text{hot}} = +3.2\ \text{mV}$$

In the main scenario, the CRS effect was operationally represented exclusively by γ_{rh}. Data regarding E_{GABA} shifts and KCC2 impairment were retained as mechanistic background of the stress module but were not inserted separately into the final calculations.

S4.3. Order of Module Application

First, the baseline static margin is reduced by the inflammatory component:

$$\Delta V_{\text{margin,1}} = \Delta V_{\text{margin,0}} - \Delta V_{\text{rest,infl,stress}}$$

Next, stress-induced excitability compresses the remaining margin:

$$\Delta V_{\text{eff,stress}} = \gamma_{\text{rh}} \cdot \Delta V_{\text{margin,1}}$$

Finally, the transient "hot spot" module produces an additional loss of margin:

$$\Delta V_{\text{eff,final}} = \Delta V_{\text{eff,stress}} - \Delta V_{\text{hot}}$$

S4.4. Final Working Equation

Combining all three modules yields:

$$\Delta V_{\text{eff,final}} = \gamma_{\text{rh}}\left(\Delta V_{\text{margin,0}} - \Delta V_{\text{rest,infl,stress}}\right) - \Delta V_{\text{hot}}$$

Substituting main scenario parameters:

$$\Delta V_{\text{eff,final}} = 0.56(18.4 - 2.0) - 3.2$$
$$\Delta V_{\text{eff,final}} = 0.56 \times 16.4 - 3.2$$
$$\Delta V_{\text{eff,final}} = 9.184 - 3.2 = 5.984\ \text{mV}$$

which, after rounding, gives:

$$\Delta V_{\text{eff,final}} \approx 6.0\ \text{mV}$$

S4.5. Total Margin Loss

Total margin narrowing relative to baseline was defined as:

$$\Delta V_{\text{loss,total}} = \Delta V_{\text{margin,0}} - \Delta V_{\text{eff,final}}$$

Substituting values:

$$\Delta V_{\text{loss,total}} = 18.4 - 5.984 = 12.416\ \text{mV}$$

which, after rounding, gives:

$$\Delta V_{\text{loss,total}} \approx 12.4\ \text{mV}$$

Equivalently, after algebraic simplification:

$$\Delta V_{\text{loss,total}} = (1 - \gamma_{\text{rh}})\Delta V_{\text{margin,0}} + \gamma_{\text{rh}}\Delta V_{\text{rest,infl,stress}} + \Delta V_{\text{hot}}$$

For the main scenario:

$$\Delta V_{\text{loss,total}} = 0.44 \times 18.4 + 0.56 \times 2.0 + 3.2$$
$$\Delta V_{\text{loss,total}} = 8.096 + 1.12 + 3.2 = 12.416\ \text{mV}$$

S4.6. Model Scope

The present model does not quantitatively incorporate additional mechanisms that could further narrow the excitability margin, such as changes in GIRK/leak conductance

contributions or additional cytokine-dependent alterations of chloride transport. These mechanisms were not included due to the absence of directly transferable numerical parameters specific to vCA1. Their exclusion should therefore be interpreted as a conservative element of the model.

S4.7. Illustrative Local Sensitivity Analysis of the Main Scenario

To assess whether the main conclusion depends on one exact point estimate, we performed an illustrative local sensitivity analysis around the main scenario. This was not intended as a formal global uncertainty propagation, but rather as a simple robustness check within the phenomenological framework.

Using the working equation

$$\Delta V_{\mathrm{eff,final}} = \gamma_{\mathrm{rh}}\left(\Delta V_{\mathrm{margin,0}} - \Delta V_{\mathrm{rest,infl,stress}}\right) - \Delta V_{\mathrm{hot}}$$

we varied each of the three main scenario parameters one at a time around the adopted reference values while holding the remaining parameters constant.

First, varying the stress-compression term γ_{rh} from 0.50 to 0.62 yielded:

$$\gamma_{\mathrm{rh}} = 0.50 \ \rightarrow \ \Delta V_{\mathrm{eff,final}} = 5.0\ \mathrm{mV}$$

$$\gamma_{\mathrm{rh}} = 0.62 \ \rightarrow \ \Delta V_{\mathrm{eff,final}} = 7.0\ \mathrm{mV}$$

Second, varying the inflammatory component $\Delta V_{\mathrm{rest,infl,stress}}$ from 1.0 to 3.0 mV yielded:

$$\Delta V_{\mathrm{rest,infl,stress}} = 1.0\ \mathrm{mV} \ \rightarrow \ \Delta V_{\mathrm{eff,final}} = 6.5\ \mathrm{mV}$$

$$\Delta V_{\mathrm{rest,infl,stress}} = 3.0\ \mathrm{mV} \ \rightarrow \ \Delta V_{\mathrm{eff,final}} = 5.4\ \mathrm{mV}$$

Third, varying the transient hot-spot component ΔV_{hot} from 2.5 to 4.0 mV yielded:

$$\Delta V_{\mathrm{hot}} = 2.5\ \mathrm{mV} \ \rightarrow \ \Delta V_{\mathrm{eff,final}} = 6.7\ \mathrm{mV}$$

$$\Delta V_{\mathrm{hot}} = 4.0\ \mathrm{mV} \ \rightarrow \ \Delta V_{\mathrm{eff,final}} = 5.2\ \mathrm{mV}$$

Finally, combining a more conservative parameter set $\gamma_{\mathrm{rh}} = 0.50, \Delta V_{\mathrm{rest,infl,stress}} = 3.0\ \mathrm{mV}, \Delta V_{\mathrm{hot}} = 4.0\ \mathrm{mV}$ yielded:

$$\Delta V_{\mathrm{eff,final}} = 3.7\ \mathrm{mV}$$

whereas a less severe combined set $\gamma_{\mathrm{rh}} = 0.62, \Delta V_{\mathrm{rest,infl,stress}} = 1.0\ \mathrm{mV}, \Delta V_{\mathrm{hot}} = 2.5\ \mathrm{mV}$ yielded:

$$\Delta V_{\mathrm{eff,final}} = 8.3\ \mathrm{mV}$$

These calculations indicate that, across a reasonable local variation of the main phenomenological parameters, the model continues to place the system within a low-margin regime rather than returning it to a clearly wide-margin baseline state. Accordingly, the qualitative conclusion of marked excitability-margin narrowing does not depend on a single exact parameter value, although the precise numerical estimate remains model-dependent and illustrative.

Supplementary Methods 5

Additional Risk Modifiers Narrowing the Excitability Margin

S5.1. Purpose of the Module

Selected risk modifiers were additionally analyzed to estimate whether they could further reduce the excitability margin of vCA1 pyramidal neurons beyond the main scenario. Three illustrative variants were examined:

1. A stronger inflammatory component,
2. An additional depolarizing effect of caffeine,
3. A chronic pro-excitatory component modeled based on data from an APP model

S5.2. Baseline and Calculation Principles

The starting point was the result of the main scenario:

$$\Delta V_{\text{eff,final}} = 5.984 \text{ mV}$$

Three calculation modes were applied depending on the nature of the modifier.

S5.2.1. Replacement of an Existing Component of the Same Type

For the stronger inflammatory variant, the previously adopted value $\Delta V_{\text{rest,infl,stress}}$ was replaced with a new value:

$$\Delta V_{\text{eff}} = \gamma_{\text{rh}}\left(\Delta V_{\text{margin,0}} - \Delta V_{\text{infl,new}}\right) - \Delta V_{\text{hot}}$$

S5.2.2. Additional Narrowing After the Main Scenario

For an acute additional depolarizing effect, a direct subtraction of the new component from the main scenario result was applied:

$$\Delta V_{\text{eff,risk}} = \Delta V_{\text{eff,final}} - \Delta V_{\text{risk}}$$

S5.2.3. Additional Chronic Component Acting Before Stress Compression

For a chronic modifier shifting the baseline margin, the new component was introduced before application of the stress compression coefficient:

$$\Delta V_{\text{eff,risk}} = \gamma_{\text{rh}}\left(\Delta V_{\text{margin,0}} - \Delta V_{\text{infl,stress}} - \Delta V_{\text{risk}}\right) - \Delta V_{\text{hot}}$$

S5.3. Parameters Retained from the Main Scenario

Across all variants, the following main scenario parameters were retained (2,4,9,10):

$$\Delta V_{\text{margin,0}} = 18.4 \text{ mV}$$
$$\gamma_{\text{rh}} = 0.56$$
$$\Delta V_{\text{infl,stress}} = 2.0 \text{ mV}$$
$$\Delta V_{\text{hot}} = 3.2 \text{ mV}$$

S5.4. Input Parameters of the Analyzed Modifiers

S5.4.1. Stronger Inflammatory Component

As a boundary-case variant, the full LPS-like effect was modeled as:

$$\Delta V_{\text{infl,new}} = +6.0 \text{ mV}$$

This value was adopted based on approximate extraction of resting membrane potential depolarization from Fig. 2D of (4), where LPS induced a significant RMP shift without altering spike threshold (4).

S5.4.2. Caffeine Exposure

Application of 100 µM caffeine induces significant membrane depolarization in CA1 pyramidal neurons, accompanied by increased input resistance, reduction of the long afterhyperpolarization phase, and decreased spike-frequency accommodation (11). The study reported a mean RMP depolarization of 4.3 ± 1.8 mV, increased input resistance, reduction of the long afterhyperpolarization phase, and decreased spike-frequency accommodation. The examined neurons had a mean resting membrane potential of 72 ± 7.6 mV (i.e., approximately −72 mV). Thus, at 100 µM caffeine, the average RMP shifted from approximately −72.0 mV to −67.7 mV. The final value was not explicitly stated by the authors but follows from subtracting the reported depolarization from the baseline resting value.

Accordingly, the model adopted:

$$\Delta V_{\text{caff}} = +4.3 \text{ mV}$$

S5.4.3. Genetic Pro-Excitatory Component: APP Model

In the Thy1-APP model, CA1 pyramidal neurons exhibit a more depolarized resting membrane potential compared to wild-type (WT) controls (12):

WT: −61.70 ± 3.46 mV

APPtg: −58.67 ± 6.42 mV

From these values, the difference was calculated as:

$$\Delta V_{\text{APP}} = (-58.67) - (-61.70) = +3.03 \text{ mV}$$

The value +3.03 mV was treated as an additional chronic component narrowing the baseline margin prior to stress compression. This calculation was performed based on mean values reported by (12).

Supplementary Methods 6

Amplitudes of Transient Network Events Potentially Exceeding the Narrowed Excitability Margin

S6.1. Purpose of the Module

The purpose of this module was to compile quantitatively reported amplitudes of transient depolarizing events observed in pyramidal neurons (or their somatic equivalents) that, under conditions of a narrowed excitability margin (ΔV_{eff}), could—at least in voltage terms—become sufficient to exceed spike threshold.

S6.2. Operational Definition of a "Trigger"

In the present work, a trigger is defined as a transient depolarizing event with measurable amplitude ΔV_{soma} (i.e., somatic membrane potential change or the somatic equivalent of a dendritic event) whose magnitude can be directly compared to the remaining excitability margin ΔV_{eff}.

These events are not interpreted as evidence of replay per se, but rather as candidate voltage-based initiation mechanisms under conditions of reduced margin.

S6.3. Fast Events and Their Reported Amplitudes

S6.3.1. Local NMDA Spikes / NMDA Spike–Plateau Events

Schiller et al. demonstrated that local dendritic NMDA spikes in basal dendrites of cortical pyramidal neurons produce a cable-filtered somatic depolarization with a peak amplitude of 5.9 ± 1.5 mV (13).

Major et al. further showed that the somatic amplitude of NMDA spike/plateau events depends strongly on input location, ranging approximately from ~3 mV for distal inputs to ~23 mV for proximal inputs (14).

S6.3.2. Somatic Depolarization During Sharp Waves (SPW)

Valero et al. reported two distinct subthreshold response types in CA1 pyramidal neurons during sharp waves; in the subgroup exhibiting depolarizing responses, the mean peak amplitude was 4.1 ± 2.3 mV (n = 12), whereas a separate subgroup displayed hyperpolarizing responses of −2.4 ± 1.2 mV (n = 10) (15).

S6.3.3. High-Frequency "Ripple" Oscillations in Intracellular Recordings

Ylinen et al. reported that during ripple oscillations (~200 Hz), intracellular ripple amplitude ranges approximately from ~1 to 4 mV depending on membrane polarization state (16).

S6.3.4. Subthreshold Gamma Oscillations in CA1

Penttonen et al. described subthreshold membrane potential fluctuations in the gamma frequency band in CA1 with amplitudes of approximately ~0.2–2 mV at resting membrane potential (17).

S6.3.5. Large Depolarizations Following Bursts

Harvey et al. described spikelets with an amplitude of 7.4 ± 1.3 mV (FWHM 1.6 ± 0.4 ms) and reported that bursts of action potentials were followed by large depolarizations in the range of ~10–25 mV lasting approximately 50–100 ms (18).

S6.3.6. Subthreshold Depolarization Within Place Fields ("Place-Field Hills")

Epsztein et al. reported that in hippocampal place cells, subthreshold depolarization within the place field ranged from ~5 to 20 mV, with a mean value of 12.8 ± 2.8 mV (19).

S6.4. Scope of the Module

The above values were obtained from diverse experimental systems (in vitro / in vivo), brain regions, behavioral states, and anesthetic conditions. Within this module, they were treated as a catalog of physiologically reported amplitude ranges rather than as parameters specific exclusively to vCA1.

The sole purpose of this module was to compare the order of magnitude of ΔV_{soma} with ΔV_{eff}. No additional modeling of channel dynamics, inhibitory strength, temporal synchronization, or network architecture was introduced.

References

1. Doyon N, Prescott SA, Castonguay A, Godin AG, Kröger H, De Koninck Y. Efficacy of Synaptic Inhibition Depends on Multiple, Dynamically Interacting Mechanisms Implicated in Chloride Homeostasis. Morrison A, editor. PLoS Comput Biol. 2011 Sep 8;7(9):e1002149. doi:10.1371/journal.pcbi.1002149

2. MacKenzie G, Maguire J. Chronic stress shifts the GABA reversal potential in the hippocampus and increases seizure susceptibility. Epilepsy Res. 2015 Jan;109:13–27. doi:10.1016/j.eplepsyres.2014.10.003

3. Dougherty KA, Islam T, Johnston D. Intrinsic excitability of CA1 pyramidal neurones from the rat dorsal and ventral hippocampus. J Physiol. 2012 Nov 15;590(22):5707–22. doi:10.1113/jphysiol.2012.242693

4. Cheng J, Zheng B, Luo S, Yuan Y, Wu X, Jiang Z, et al. Argon improves microglia-mediated hippocampal neuronal hyperexcitability to alleviate anxiety-like behaviors in mice. Luo ZG, editor. J Mol Cell Biol. 2025 Nov 11;17(4):mjaf006. doi:10.1093/jmcb/mjaf006

5. Calcia MA, Bonsall DR, Bloomfield PS, Selvaraj S, Barichello T, Howes OD. Stress and neuroinflammation: a systematic review of the effects of stress on microglia and the implications for mental illness. Psychopharmacology (Berl). 2016 May;233(9):1637–50. doi:10.1007/s00213-016-4218-9

6. Guo T, Guo Z, Yang X, Sun L, Wang S, Yingge A, et al. The Alterations of IL-1Beta, IL-6, and TGF-Beta Levels in Hippocampal CA3 Region of Chronic Restraint Stress Rats after Electroacupuncture (EA) Pretreatment. Lin YW, editor. Evid Based Complement Alternat Med. 2014 Jan;2014(1):369158. doi:10.1155/2014/369158

7. Pignatelli M, Ryan TJ, Roy DS, Lovett C, Smith LM, Muralidhar S, et al. Engram Cell Excitability State Determines the Efficacy of Memory Retrieval. Neuron. 2019 Jan;101(2):274-284.e5. doi:10.1016/j.neuron.2018.11.029

8. Jimenez JC, Berry JE, Lim SC, Ong SK, Kheirbek MA, Hen R. Contextual fear memory retrieval by correlated ensembles of ventral CA1 neurons. Nat Commun. 2020 Jul 13;11(1):3492. doi:10.1038/s41467-020-17270-w

9. Zhou Y, Won J, Karlsson MG, Zhou M, Rogerson T, Balaji J, et al. CREB regulates excitability and the allocation of memory to subsets of neurons in the amygdala. Nat Neurosci. 2009 Nov;12(11):1438–43. doi:10.1038/nn.2405

10. Cembrowski MS, Bachman JL, Wang L, Sugino K, Shields BC, Spruston N. Spatial Gene-Expression Gradients Underlie Prominent Heterogeneity of CA1 Pyramidal Neurons. Neuron. 2016 Jan;89(2):351–68. doi:10.1016/j.neuron.2015.12.013

11. Greene RW, Haas HL, Hermann A. Effects of caffeine on hippocampal pyramidal cells *in vitro*. Br J Pharmacol. 1985 May;85(1):163–9. doi:10.1111/j.1476-5381.1985.tb08843.x

12. Sosulina L, Mittag M, Geis H, Hoffmann K, Klyubin I, Qi Y, et al. Hippocampal hyperactivity in a rat model of Alzheimer's disease. J Neurochem. 2021 Jun;157(6):2128–44. doi:10.1111/jnc.15323

13. Schiller J, Major G, Koester HJ, Schiller Y. NMDA spikes in basal dendrites of cortical pyramidal neurons. Nature. 2000 Mar;404(6775):285–9. doi:10.1038/35005094

14. Major G, Polsky A, Denk W, Schiller J, Tank DW. Spatiotemporally Graded NMDA Spike/Plateau Potentials in Basal Dendrites of Neocortical Pyramidal Neurons. J Neurophysiol. 2008 May;99(5):2584–601. doi:10.1152/jn.00011.2008

15. Valero M, Cid E, Averkin RG, Aguilar J, Sanchez-Aguilera A, Viney TJ, et al. Determinants of different deep and superficial CA1 pyramidal cell dynamics during sharp-wave ripples. Nat Neurosci. 2015 Sep;18(9):1281–90. doi:10.1038/nn.4074

16. Ylinen A, Bragin A, Nadasdy Z, Jando G, Szabo I, Sik A, et al. Sharp wave-associated high-frequency oscillation (200 Hz) in the intact hippocampus: network and intracellular mechanisms. J Neurosci. 1995 Jan 1;15(1):30–46. doi:10.1523/JNEUROSCI.15-01-00030.1995

17. Penttonen M, Kamondi A, Acsády L, Buzsáki G. Gamma frequency oscillation in the hippocampus of the rat: intracellular analysis *in vivo*. Eur J Neurosci. 1998 Feb;10(2):718–28. doi:10.1046/j.1460-9568.1998.00096.x

18. Harvey CD, Collman F, Dombeck DA, Tank DW. Intracellular dynamics of hippocampal place cells during virtual navigation. Nature. 2009 Oct;461(7266):941–6. doi:10.1038/nature08499

19. Epsztein J, Brecht M, Lee AK. Intracellular Determinants of Hippocampal CA1 Place and Silent Cell Activity in a Novel Environment. Neuron. 2011 Apr;70(1):109–20. doi:10.1016/j.neuron.2011.03.006
